# VEGF-dependent testicular vascularisation involves MEK1/2 signalling and the essential angiogenesis factors, SOX7 and SOX17

**DOI:** 10.1186/s12915-024-02003-y

**Published:** 2024-10-01

**Authors:** Rheannon O. Blücher, Rachel S. Lim, Matthew E. Ritchie, Patrick S. Western

**Affiliations:** 1 Centre for Reproductive Health, Hudson Institute of Medical Research and Department of Molecular and Translational Science, Monash University, Clayton, VIC 3168 Australia; 2 Epigenetics and Development Division, The Walter and Eliza Hall Institute of Medical Research, Parkville, VIC 3052 Australia

**Keywords:** Testis development, Vasculature, VEGF, SOX7, SOX17, MAP kinase, MEK1/2, FGF, Sertoli cells, Endothelial cells

## Abstract

**Background:**

Abnormalities of *in utero* testis development are strongly associated with reproductive health conditions, including male infertility and testis cancer. In mouse testes, SOX9 and FGF9 support Sertoli cell development, while VEGF signalling is essential for the establishment of vasculature. The mitogen-activated protein kinase (MAPK) pathway is a major signalling cascade, essential for cell proliferation, differentiation and activation of *Sry* during primary sex-determination, but little is known about its function during fetal testis morphogenesis. We explored potential functions of MAPK signalling immediately after the establishment of testis cords in embryonic day (E)12.5 *Oct4*-eGFP transgenic mouse testes cultured using a MEK1/2 inhibitor.

**Results:**

RNA sequencing in isolated gonadal somatic cells identified 116 and 114 differentially expressed genes after 24 and 72 h of MEK1/2 inhibition, respectively. Ingenuity Pathway Analysis revealed an association of MEK1/2 signalling with biological functions such as angiogenesis, vasculogenesis and cell migration. This included a failure to upregulate the master transcriptional regulators of vascular development, *Sox7* and *Sox17,* VEGF receptor genes*,* the cell adhesion factor gene *Cd31* and a range of other endothelial cell markers such as *Cdh5* (encoding VE-cadherin) and gap junction genes *Gja4* and *Gja5*. In contrast, only a small number of Sertoli cell enriched genes were affected. Immunofluorescent analyses of control testes revealed that the MEK1/2 downstream target, ERK1/2 was phosphorylated in endothelial cells and Sertoli cells. Inhibition of MEK1/2 eliminated pERK1/2 in fetal testes, and CD31, VE-cadherin, SOX7 and SOX17 and endothelial cells were lost. Consistent with a role for VEGF in driving endothelial cell development in the testis, inhibition of VEGFR also abrogated pERK1/2 and SOX7 and SOX17 expressing endothelial cells. Moreover, while Sertoli cell proliferation and localisation to the testis cord basement membrane was disrupted by inhibition of MEK1/2, it was unaffected by VEGFR inhibition. Instead, inhibition of FGF signalling compromised Sertoli cell proliferation and localisation to the testis cord basement membrane.

**Conclusions:**

Together, our data highlight an essential role for VEGF-dependent MEK1/2 signalling in promoting vasculature and indicate that FGF signalling through MEK1/2 regulates Sertoli cell organisation in the developing mouse testis.

**Supplementary Information:**

The online version contains supplementary material available at 10.1186/s12915-024-02003-y.

## Background

Formation of a properly functioning testis is essential for endocrine regulation, spermatogenesis and male fertility with abnormalities in testis development *in utero* strongly associated with abnormal steroidogenesis, male infertility, and testis cancer [1, 2]. Testis morphogenesis is a highly coordinated process, involving several cell types including Sertoli, germ, interstitial, endothelial and immune cells. At embryonic day (E)10.5, initiation of *Sry* (sex-determining region Y) expression in pre-Sertoli cells promotes *Sox9* (SRY box 9) expression and drives commitment to Sertoli cell fate [3]. At E11.5, XY and XX gonads exhibit similar vasculature, with a vasculature plexus present at the gonad mesonephric border [4]. Testis cords begin to develop in response to the expression of *Sry*, *Sox9* and *Fgf9* (fibroblast growth factor 9) [5]. In XY mice, SRY activates *Sox9* expression, and SOX9 drives FGF signalling to ensure proper cord formation and the proliferation of Sertoli cells in the developing testis [6–13]. The mesonephric vascular plexus is broken down between E11.5 and E12.5, and endothelial cells begin to migrate into the testes [4, 14]. The migration of endothelial cells divides the testis into regions with and without vasculature, the latter of which contain developing testis cords [4, 14]. During this period, endothelial cells migrate towards the coelomic domain and coalesce to form the coelomic vessel [4, 14].

Vascular endothelial growth factor (VEGF) signalling plays an essential role in vascularisation of many tissues in the embryo, including the testis. In the fetal mouse testis, VEGFA is highly expressed in interstitial cells at E12.5 [15]. Inhibition of VEGF in the whole gonad or specifically in the vasculature of E11.5 XY gonads reduced the number of endothelial cells and prevented testis cord formation [15, 16]. However, Sertoli and 3β-HSD (3β-hydroxysteroid dehydrogenase) positive Leydig cells were still detected, although the latter were detected throughout the gonad rather than being properly localised within the interstitium [15]. While disrupting VEGF signalling at E11.5 affected both vascular and testicular structure, inhibition of VEGF from E12.5 prevented vasculature development but did not impact testis cord structure, permitting the study of vascularisation in the presence of testis cords [16]. Together, these studies highlight an essential role for VEGF signalling for vasculature patterning in the fetal testis but the downstream mechanisms by which this occurs are not understood.

The SOXF family of transcription factors, comprising of SOX7, SOX17, and SOX18, play essential roles in vasculature development in the embryo [17–19]. Global deletion of *Sox7* or conditional deletion of *Sox7* in *Vegfr2* (VEGF receptor 2) or *Tie2* (TEK receptor tyrosine kinase) expressing cells resulted in embryonic lethality by E10.5–12.5 [20–22]. These early embryos exhibited failures in vasculature development and/or poor vasculature remodelling, highlighting an essential role for *Sox7* in endothelial cells in promoting vasculogenesis and angiogenesis [21, 22]. Similarly, global and conditional deletion of *Sox17* in endothelial precursors or endothelial cells result in embryonic lethality by E10.5–12.5 or disrupted vasculature development and remodelling [22–25]. *Sox18* expression has been identified in vessels such as the dorsal aorta and intersomitic vessels in mice [26]. Moreover, defects in cardiovascular system of *ragged* mice have been attributed to *Sox18* mutations [26, 27] and redundancy has been observed between *Sox7, Sox17* and/or *Sox18* in a range of vascular developmental contexts [21, 25, 28–32]. Since *SoxF* genes promote angiogenesis in the early embryo, it is plausible that these genes may also play a role in vasculature patterning in the fetal testis.

The mitogen-activated protein kinase (MAPK) pathway is a major signalling cascade responsible for regulating processes including cell proliferation, differentiation, and apoptosis. Growth factors and mitogens commonly activate the MEK/ERK (mitogen-activated protein kinase kinase/extracellular signal-regulated kinase) branch of the MAPK family to promote cell differentiation in a range of developmental contexts. For example, VEGFA activates ERK1/2 to promote intersomitic vessel angiogenesis in zebrafish [33] and PDGF-AA (platelet-derived growth factor AA) activates ERK in Leydig cells to promote proliferation and migration in the adult mouse testis [34]. MAPK signalling also affects primary sex determination in mice and testis development in humans. Deletion of *Map3k4* or *Map3k6* results in reduced *Sry* expression and male to female gonadal sex reversal [35], while loss of *Map2k3* results in disrupted testis development and the formation of ovotestes [36]. Moreover, mutations in *MAP3K1* are relatively common in differences of sex development (DSD) patients with 46, XY complete gonad dysgenesis [37]. However, while *Map3k1* is also expressed in mouse gonads at E11.5, deletion in XY mice resulted only in increased gonadal length [38]. MEK1/2 signalling has also been detected in Sertoli cells and within cells of the interstitial space of developing fetal testes [13]. Moreover, inhibition of MEK1/2 from E12.5 resulted in reduced Sertoli cell proliferation and disrupted germline differentiation, indicating that MEK1/2 signalling may also regulate testis development after primary sex determination [13], a possibility that has not been studied.

In this study, we explored the impact of MEK1/2 signalling inhibition in E12.5 mouse testes. Our data demonstrate that MEK1/2 signalling is essential for vasculature patterning in the testis and localisation of Sertoli cell to the testis cord basement membrane from E12.5. Furthermore, testicular vascularisation is dependent on VEGF signalling as VEGF inhibition reduced phospho-ERK1/2 (pERK1/2) and SOX7/17 in endothelial cells, and prevented normal vascular organisation of endothelial cells. Together, our data reveals a VEGF-dependent signalling cascade that acts via MEK1/2 to promote testicular angiogenesis and is required for SOX7 and SOX17 expressing endothelial cells in the developing testis. In addition, we identify an essential role for MEK1/2 signalling in organisation of Sertoli cells in the testis cords that is independent of VEGF signalling.

## Results

### MEK1/2 signalling is essential for the expression of endothelial specific genes

**Fig. 1:**
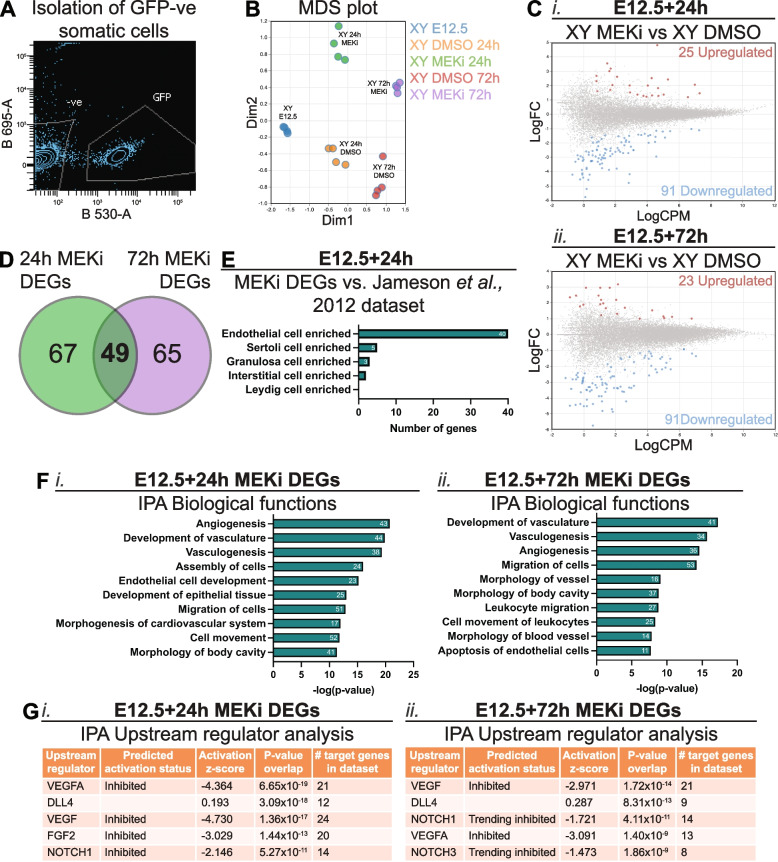
MEK1/2 signalling is required for expression of genes associated with endothelial cells, vasculogenesis and angiogenesis in somatic cells of developing testes. RNA sequencing analysis of somatic cells isolated from E12.5 testes and E12.5 testes cultured for 24 or 72h with DMSO or 500nM of MEKi. **A** Example of FACS scatterplot depicting isolation of GFP negative somatic cells. **B** Multidimensional scaling (MDS) plot of somatic cell samples subject to RNA sequencing. **C** Differential gene expression analysis of E12.5 + 24 h (i) or E12.5 + 72 h (ii) MEKi vs DMSO. Genes with FDR < 0.05 and |logFC|≥ 0.585 (equivalent to |FC|≥ 1.5) were considered differentially expressed. **D** Venn Diagram comparing somatic cell RNA sequencing data from E12.5 testis samples cultured with DMSO or MEKi 24h and 72h to identify common differentially expressed genes (DEGs). **E** Comparison of 24h MEKi DEGs with the microarray data set published by Jameson et al., [44], PLOS Genetics. **F** Ingenuity Pathway Analysis (IPA) of biological functions associated with 24h (i) and 72h (ii) MEKi DEGs. The number of genes associated with each term is shown within the bar. **G** IPA of upstream regulators associated with 24h (i) and 72h (ii) MEKi DEGs. Biological replicates; *n* = 4 per treatment

We previously demonstrated that MEK1/2 inhibition disrupts Sertoli cell proliferation and organisation [13]. To investigate the role of MEK1/2 signalling in the somatic cells of developing testis further, *Oct4 (Pou5f1)*-eGFP (OG2) transgenic male mice (129T2svJ background) [39–41] were crossed to Swiss females and E12.5 testis-mesonephros samples were isolated and cultured for 24 or 72h with DMSO or the small molecule inhibitor PD0325901/Mirdametinib (MEKi) at a concentration of 500nM. This dose was selected based on previous titration experiments that identified a minimal dose that functionally altered germ cell mitotic arrest and Sertoli cell proliferation, and completely abrogated the ability of FGF9 to promote somatic cell proliferation in E12.5 XX gonads [13]. To gain insight into genome-wide transcriptional changes that occur in the somatic cells of MEK1/2 inhibited testes, RNA sequencing was performed on testicular somatic cells obtained by fluorescent activated cell sorting (FACS) which isolated *Oct4*-GFP negative cells of E12.5 XY gonads cultured for 24 or 72h (Fig. 1A; [40, 41]. The RNA sequencing data from each treatment group clustered independently in a multidimensional scaling plot (Fig. 1B), indicating that inhibition of MEK1/2 signalling altered somatic cell transcription. Further analysis identified 116 and 114 differentially expressed genes (DEGs) following culture in MEKi for 24 or 72h (Additional file 1: Table S1.1 and S1.2), respectively (false discovery rate (FDR) < 0.05, absolute fold-change (FC) ≥ 1.5, absolute log-FC ≥ 0.585; Fig. 1C). Comparison of the DEGs identified 49 genes which were commonly disrupted after 24 or 72h of MEKi (Fig. 1D; Additional file 1: Table S1.3). These included endothelial associated genes such as *Pecam1* (*Cd31*), *Flt1* (*Vegfr1*), *Ecscr*, and *Tie1*, the angiogenesis marker *Sox7*, and the cell-surface glycoprotein encoding gene *Cd93,* which has been associated with enhanced macrophage phagocytosis and inter-cellular adhesion [42]. In addition, 67 genes were uniquely disrupted after 24h of MEKi (Fig. 1D; Additional file 1: Table S1.3) including the gap junction genes *Gja4* and *Gja5*, the angiogenesis marker *Sox17*, and the chemokine receptor *Cxcr4*. Similarly, MEK1/2 inhibition for 72h exclusively disrupted 65 genes (Fig. 1D; Additional file 1: Table S1.3), including the retinoic acid degrading enzyme coding gene *Cyp26b1* and the type IV collagen coding genes *Col4a1 and Col4a2*, which function downstream of *Wt1* to support testis cord integrity in the fetal mouse testis [43].

The somatic cell population of the fetal testis and ovary is comprised of supporting cells (Sertoli – XY and granulosa – XX) which surround germ cells in the XY fetal gonad, stromal (XX) and interstitial (XY) cells which give rise to steroidogenic theca (XX) and Leydig (XY) cells, endothelial cells, which contribute to vasculature and cord formation in XY gonads, and immune cells [44]. To determine if particular cell populations were affected by MEKi more than others, we compared our MEKi DEGs with published data [44]. As Jameson et al., [44] assessed E11.5–13.5 gonads and our samples are most closely aligned in age with E13.5 gonads, we compared our 24h MEKi DEGs with the E13.5 data. Of the 116 DEGs identified, 40 were specifically enriched in endothelial cells, five in Sertoli cells, three in granulosa cells and two in interstitial cells at E13.5 (Fig. 1E, Additional file 1: Table S1.4). All 40 genes specifically enriched in endothelial cells were down regulated after 24h MEKi treatment indicating either that endothelial cells are lost, that transcription of these genes depends on MEK1/2 signalling or both (Additional file 1: Table S1.4). The remaining genes were not specifically enriched in any cell type (46 genes) or were not detected in the microarray data set (20 genes; Additional file 1: Table S1.4).

To determine the biological functions affected by MEKi, we assessed the 24h and 72h MEKi DEGs using Ingenuity Pathway Analysis (IPA; Fig. 1F). IPA determined that many 24 h MEKi DEGs were associated with angiogenesis (43 genes), development of vasculature (44 genes), and vasculogenesis (38 genes), assembly of cells (24 genes) and migration of cells (51 genes; Fig. 1Fi). Some of these terms were also associated with MEKi 72h DEGs including, development of vasculature (41 genes), vasculogenesis (34 genes), angiogenesis (36 genes), and migration of cells (53 genes; Fig. 1Fii). Other terms including morphology of vessel (16 genes) and apoptosis of endothelial cells (11 genes) were also associated with MEKi 72h DEGs. Although terms relating to cell death or apoptosis were not present in the top 10 most associated biological functions in the 24h MEKi DEGs, cell death of endothelial cells was the 27th associated term, while apoptosis was 29th and apoptosis of endothelial cells was 31st. Moreover, IPA upstream regulator analysis predicted upstream pathways associated with the 24h MEKi DEGs including VEGFA, DLL4, VEGF, FGF2 and NOTCH1 (Fig. 1Gi). Unsurprisingly, the top five predicted upstream regulators of the 72h MEKi DEGs were similar to the 24h MEKi DEGs and included VEGF, DLL4, NOTCH1, VEGFA and NOTCH3 (Fig. 1Gii). Together these analyses highlight very strong endothelial cell, angiogenesis and vasculogenesis signatures in testes following MEK1/2 inhibition, indicating that MEK1/2 may act downstream of VEGFA/VEGF signalling to promote the expansion and organisation of the fetal testis vasculature.

### MEK1/2 signalling is essential for endothelial cell development in fetal testes

To determine if MEK1/2 signalling acts directly in endothelial cells, we used IF to assess the localisation of the downstream target of MEK1/2, pERK1/2 and the endothelial cell marker, CD31, in E12.5, E13.5, E14.5 and E15.5 testes (Additional file 2: Fig. S1). At E12.5, strong pERK1/2 staining was detected in CD31 positive endothelial cells but not in germ cells, which had weaker CD31 staining and were clearly morphologically distinguishable from endothelial cells. Although less intense than the staining observed in endothelial cells, pERK1/2 was also detected in cells immediately surrounding germ cells. Given their punctate DAPI staining (marking DNA) and the location of these somatic cells inside testis cords, we concluded that these were Sertoli cells. Intense pERK1/2 staining was maintained in endothelial cells in E13.5 testes, but pERK1/2 was no longer detected in Sertoli cells. At E14.5, pERK1/2 was detected in endothelial cells and was again detected in Sertoli cells. Furthermore, bright pERK1/2 staining was detected in Sertoli cells located in the middle of the testis cords, and less intense staining was detected in Sertoli cells towards the testis cord basement membrane. At E15.5, endothelial cells were pERK1/2 positive and the staining in Sertoli cells remained similar to that detected in E14.5 Sertoli cells.

**Fig. 2:**
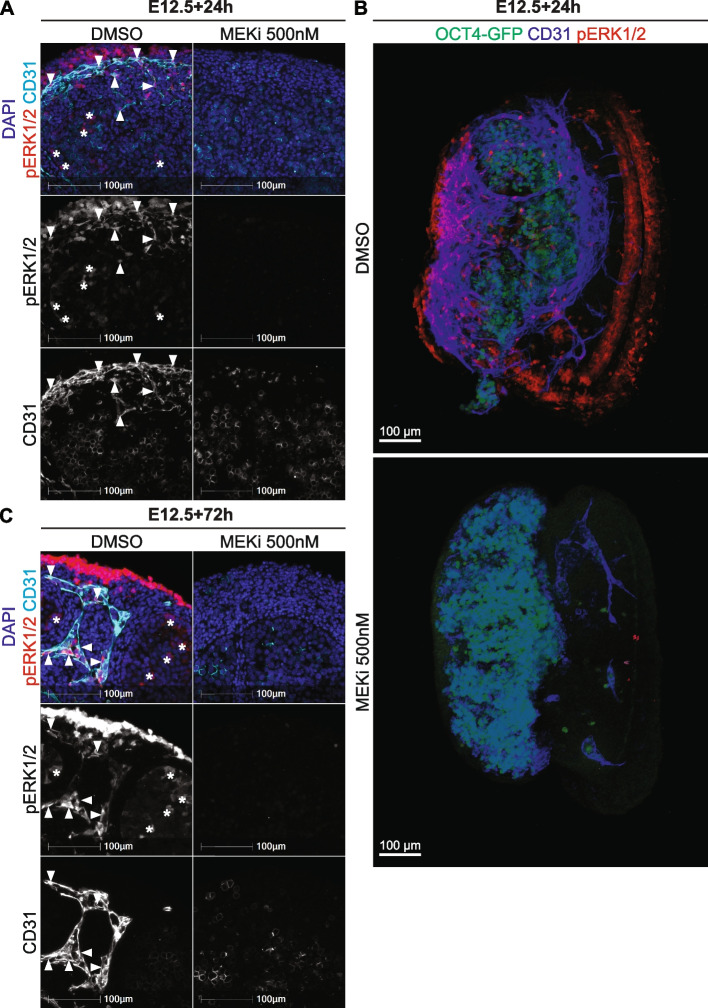
MEK1/2 inhibition depletes pERK1/2 and CD31 expressing endothelial cells in developing testes. Section or wholemount immunofluorescent imaging of E12.5 testes cultured for 24 or 72h with DMSO or 500nM of MEKi. **A** and **C** Section IF of cultured testes stained with DAPI (blue), pERK1/2 (red) and CD31 (endothelial cells and germ cells; cyan). **B** Three-dimensional stacked Z-series images of cultured testes showing *Oct4*-eGFP (germ cells; green), CD31 (endothelial cells and germ cells; blue) and pERK1/2 (red). The DMSO and MEKi images should be viewed in conjunction with Additional files 4–7: Movies S1-4, which reveal the 3D organisation of the cells in the tissue. Scale bar represents 100 μm. Arrows indicate pERK1/2 positive endothelial cells and asterisks indicate pERK1/2 positive Sertoli cells. Biological replicates; *n* = 3–4 per treatment

To confirm that MEKi inhibited MEK1/2 activity, we examined pERK1/2 localisation in E12.5 testes cultured for 24h or 72h in DMSO or MEKi media. While pERK1/2 was detected in DMSO controls, no pERK1/2 was detected in testes following 24h or 72h MEKi treatment (Fig. 2, Additional file 3: Fig. S2, Additional files 4–7: Movies S1-4). In 24h DMSO controls, pERK1/2 was detected in the CD31 positive endothelial cells, with strong pERK1/2 staining in cells of the coelomic vessel (Fig. 2A, Additional file 3: Fig. S2Ai). Moreover, weaker pERK1/2 staining was detected in Sertoli cells located in testis cords and surrounding germ cells (Fig. 2A, Additional file 3: Fig. S2Ai). Co-staining pERK1/2 with SOX9 confirmed this observation, with pERK1/2 detected in the nucleus or cytoplasm of some Sertoli cells, but not detected in other Sertoli cells possibly depending on their cell cycle state (Additional file 3: Fig. S2B). Notably, while pERK1/2 positive Sertoli cells were detected in E12.5 testes cultured for 24h (Additional file 3: Fig. S2B), pERK1/2 was not detected in E13.5 in vivo collected testes, although it was detected at E12.5 and E14.5 (Additional file 2: Fig. S1). This difference in pERK1/2 detection in E13.5 in vivo and E12.5 + 24h cultured testes is presumably due to slightly delayed development caused by isolation of E12.5 gonads and setting them in culture, consistent with transient loss of pERK1/2 in Sertoli cells between E12.5 and E13.5 (Additional file 2: Fig. S1).

To gain more detailed visualisation of endothelial cells in three-dimensional space, we cultured gonads for 24h with DMSO or MEKi in hanging drops and used wholemount IF to examine CD31 and pERK1/2. Wholemount IF revealed a similar pattern of *Oct4*-eGFP, pERK1/2 and CD31 in E12.5 testes cultured with DMSO, with pERK1/2 detected in CD31 positive endothelial cells particularly towards to coelomic epithelium, in between the testis cords and in the gonad-mesonephric border (Fig. 2B, Additional files 4–5: Movie S1-2). In addition, *Oct4*-eGFP negative Sertoli cells detected within the testis cords were pERK1/2 positive (Fig. 2B, Additional files 4–5: Movies S1-2). In contrast, no pERK1/2 positive cells were detected in 24h MEKi treated testes using section IF (Fig. 2A; Additional file 3: Fig. S2Ai). Furthermore, CD31 positive endothelial cells were not detected in these gonads indicating that vasculature was lost within the testis, although some CD31 positive endothelial cells were detected in the mesonephros (Fig. 2A, Additional file 3: Fig. S2A). This was confirmed with wholemount IFs of MEKi treated testes, with no pERK1/2 detected and the number of CD31 positive endothelial cells within the testes greatly reduced, although some endothelial cells were detected in the mesonephros (Fig. 2B, Additional files 6–7: Movies S3-4). Consistent with these findings, pERK1/2, CD31 double positive endothelial cells marked the coelomic vessel in 72h DMSO cultures (Fig. 2C, Additional file 3: S2Aii). In addition, pERK1/2 was detected in Sertoli cells, although the staining intensity was weaker than in endothelial cells (Fig. 2C, Additional file 3: Fig. S2Aii). In contrast, pERK1/2 was not detected, and CD31 positive endothelial cells were not observed in testes following 72h of culture with MEKi (Fig. 2C, Additional file 3: Fig. S2Aii). This was consistent with 3.99- and 5.94-fold reduced *Cd31* transcription observed by RNA sequencing after 24 and 72h MEKi treatment respectively (Additional file 1: Table S1.1 and S1.2), confirming that MEKi reduced both transcription and protein levels of CD31. While it is possible that MEK1/2 inhibition resulted only in loss of CD31 expression and endothelial cells remained, RNA sequencing analysis revealed lower transcriptional levels of all 40 DEGs that were endothelial cell enriched genes, strongly indicating that endothelial cells were lost (Fig. 1E; Additional file 1: Table 1.4). Moreover, IF staining for the definitive endothelial marker VE-Cadherin specifically marked well-organised endothelial cells in DMSO controls after 24h of culture, but no VE-Cadherin positive cells remained within the gonad of MEKi-treated samples (Additional file 8: Fig. S3). This was consistent with 6.7-fold reduction in *Cdh5* (encodes VE-cadherin) transcription after 24h MEK1/2 inhibition observed by RNA sequencing (Additional file 1: Table 1.1). Together these data indicate that inhibiting MEK1/2 signalling severely impacted vascular development in E12.5 testes.

### MEK1/2 inhibition results in loss of the angiogenic transcription factors SOX7 and SOX17 in testicular endothelial cells

*Sox7* and *Sox17* are genes in the *SoxF* subgroup that regulate angiogenesis and vasculogenesis [21, 25, 28–32], but their roles in the fetal testis are unknown. To define the protein expression and localisation of SOX7 and SOX17 during testis development, IF was used to examine SOX7 and SOX17 in testes collected from E12.5 and E15.5 embryos. SOX7 and SOX17 were both detected in CD31 positive endothelial cells within E12.5 and E15.5 testis, identifying them as definitive endothelial cell markers in the testis (Additional file 9: Fig. S4). Since SOX7 and SOX17 were co-expressed in CD31 positive endothelial cells and are known to act together in mediating vascular development in other tissues [32], the SOX7 and SOX17 antibodies were used in combination in subsequent experiments to increase sensitivity and ensure all SOX7/17 positive cells were detected. We next investigated E12.5, E13.5, E14.5, and E15.5 testes and found that SOX7/17 were detected in CD31 positive endothelial cells in the coelomic vessel and in endothelial cells between the testis cords (Additional file 10: Fig. S5).

**Fig. 3:**
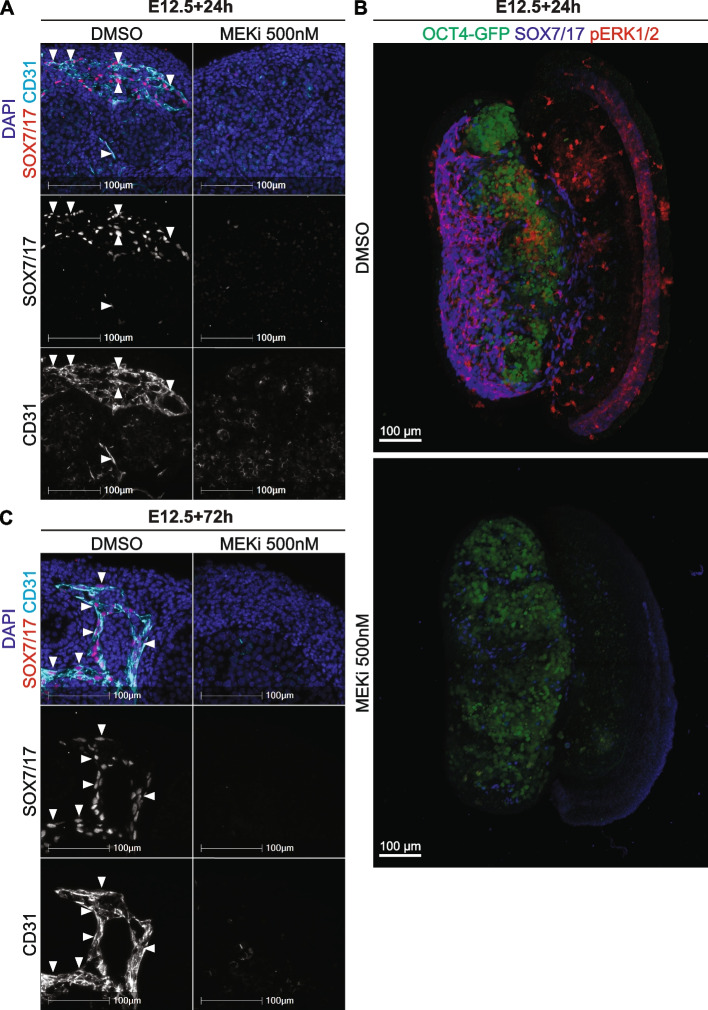
SOX7/17 expression depends on MEK1/2 signalling in the developing testis. Section or wholemount immunofluorescent imaging of E12.5 testes cultured for 24 or 72h with DMSO or 500nM of MEKi. **A** and **C** Section IF of cultured testes stained with DAPI (blue), SOX7/17 (red) and CD31 (endothelial cells and germ cells; cyan). **B** Three-dimensional stacked Z-series images of cultured testes showing *Oct4*-eGFP (germ cells; green), SOX7/17 (blue) and pERK1/2 (red). The DMSO and MEKi images should be viewed in conjunction with Movies S5-8 which reveal the 3D organisation of the cells in the tissue. Scale bar represents 100 μm. Arrows indicate SOX7/17 positive endothelial cells. Biological replicates; *n* = 3–4 per treatment

RNA sequencing revealed that *Sox7* and *Sox17* transcription were 37.5- and 9.8-fold lower in testes treated with MEKi for 24h, suggesting *Sox7* and *Sox17* depend on MEK1/2 signalling for their expression in the testis. To confirm these data and determine whether SOX7 and SOX17 proteins were reduced in MEKi-treated samples, we also assessed SOX7/17 in E12.5 24h and 72h cultures using IF. Consistent with the E12.5-E15.5 IFs, CD31 positive endothelial cells located in the coelomic vessel and the mesonephros strongly expressed SOX7/17 in DMSO-treated samples cultured for 24h (Fig. 3A, Additional file 11: Fig. S6A-B). However, consistent with the RNA sequencing data, neither SOX7/17 nor CD31 positive endothelial cells were detected in testes treated with MEKi for 24h (Fig. 3A, Additional file 11: Fig. S6A). Notably, a small number of CD31 positive cells remained in the mesonephros of MEKi-treated samples and these remaining CD31 positive cells expressed SOX7/17 at substantially lower levels than in DMSO controls (Additional file 11: Fig. S6B).

In addition, wholemount IFs of DMSO 24h controls revealed strong SOX7/17 staining co-localised with pERK1/2 in the celomic vessel, between the testis cords and in the mesonephros, but did not co-localise with *Oct4*-eGFP within the testis cords (Fig. 3B, Additional files 12–13: Movies S5-6). In 24h MEKi treated testes, pERK1/2 and SOX7/17 were not detected in the vast majority of cells, although very weak SOX7/17 staining was evident in a few remaining endothelial cells (Fig. 3B, Additional files 14–15: Movies S7-8). Together, these data suggest that SOX7 and SOX17 depend on MEK1/2 signalling in endothelial cells. Consistent with these findings, while CD31 and SOX7/17 double positive endothelial cells were detected in the coelomic vessel, between the testis cords, and in the mesonephros in 72h DMSO cultures, neither CD31, nor SOX7/17 were detected in the testis or mesonephros in 72h MEKi-treated samples (Fig. 3C, Additional file 11: Fig. S6C). Together, the RNA sequencing and IF data suggest that expression of SOX7 and SOX17 depend on MEK1/2 signalling in endothelial cells. However, it remains possible that SOX7 and SOX17 are lost simply due to endothelial cell death and further work is required to determine whether MEK1/2 signalling promotes *Sox7* and *Sox17* transcription in endothelial cells.

### MEK1/2 signalling occurs downstream of VEGF in the developing testis

**Fig. 4:**
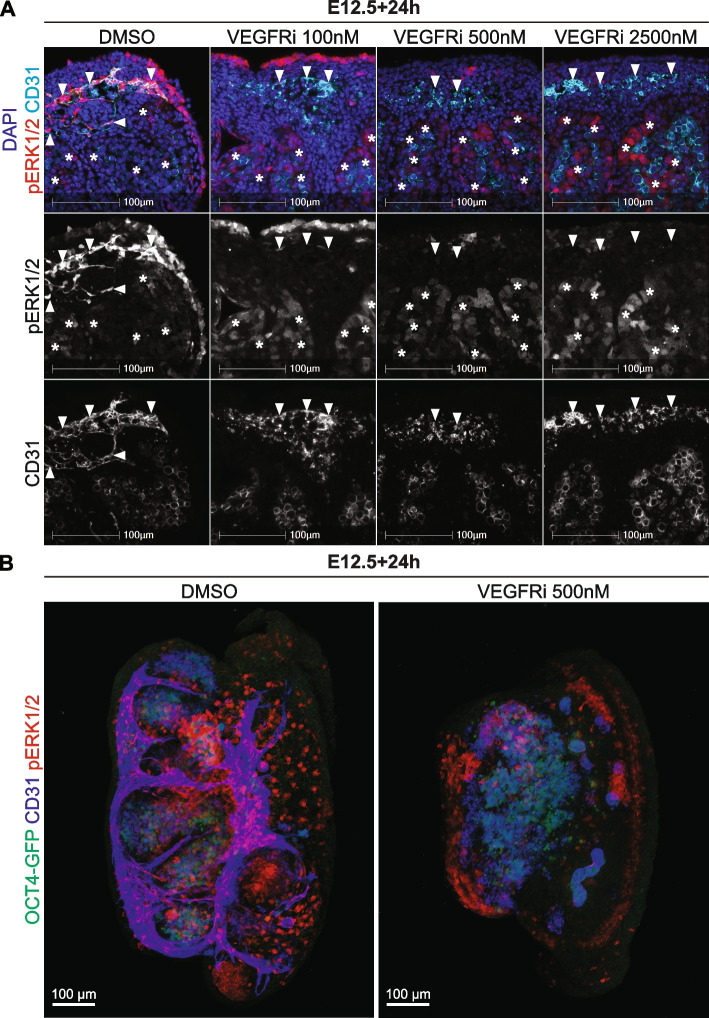
VEGFR is required for endothelial cell activation of pERK1/2 and vasculogenesis in the developing testis. Section and wholemount immunofluorescent imaging of E12.5 testes cultured for 24h with DMSO or 100, 500 or 2500 nM of VEGFRi. **A** IF in sections of cultured testes stained with DAPI (blue), pERK1/2 (red) and CD31 (endothelial cells and germ cells; cyan). **B** Three-dimensional stacked Z-series images of cultured testes showing *Oct4*-eGFP (germ cells; green), CD31 (endothelial cells and germ cells; blue) and pERK1/2 (red). The DMSO and VEGFRi images should be viewed in conjunction with Movies S9-12 which reveal the 3D organisation of the cells in the tissue. Notably, in the VEGFRi treatment this reveals a thin surface epithelium positive for pERK1/2, but negative for CD31 in the VEGFRi treated samples. Scale bar represents 100 μm. Arrows indicate endothelial cells and asterisks indicate pERK1/2 positive Sertoli cells. Biological replicates; *n* = 3–4 per treatment

VEGF signalling plays an important role in the establishment and patterning of vasculature in the testis [15]. Moreover, based on the DEGs found in our MEKi 24h and 72h RNA sequencing dataset, IPA identified VEGF/VEGFA signalling as potential upstream regulator(s) of MEK1/2 signalling in the testis. To test this, we collected E12.5 testes and cultured them for 24h in media containing 100, 500 or 2500nM of the VEGFR inhibitor, Cabozantinib (VEGFRi), and compared the outcomes with DMSO and MEKi treated testes. Using IF we assessed CD31 and pERK1/2 in endothelial cells at each dose. In the 24h DMSO control, CD31 positive endothelial cells were detected in the coelomic vessel and contained intense pERK1/2 staining (Fig. 4A, Additional file 16: Fig. S7A). In addition, consistent with our previous observations (Fig. 2, Additional file 3: Fig. S2), pERK1/2 was also detected in Sertoli cells at a lower intensity compared to endothelial cells (Fig. 4A, Additional file 16: Fig. S7A). At all VEGFRi doses, some CD31 positive endothelial cells were detected in the coelomic vessel region and the mesonephros. However, in contrast to the well organised vasculature found in DMSO controls, endothelial cells were not arranged in organised blood vessels in VEGFRi treated samples (Fig. 4A, Additional file 16: Fig. S7A), consistent with the established role for VEGRF in vascular patterning in the fetal testis. Although pERK1/2 was detected in CD31 positive endothelial cells following 100nM of VEGFRi, the intensity of pERK1/2 was substantially reduced compared to DMSO controls, indicating that 100nM VEGFRi partially inhibited VEGFR. Consistent with this, CD31 positive endothelial cells detected in gonads treated with 500 and 2500nM VEGFRi did not express pERK1/2 (Fig. 4A, Additional file 16: Fig. S7A). However, while pERK1/2 was not detected in endothelial cells, 100, 500 or 2500nM VEGFRi did not reduce pERK1/2 in Sertoli cells (Fig. 4A, Additional file 16: Fig. S7A). In addition, pERK1/2 was detected in surface epithelial cells of samples cultured with DMSO or with 100, 500 or 2500nM VEGFRi, although staining was less intense at 500 and 2500nM doses (Fig. 4A, Additional file 16: Fig. S7A). These data were confirmed in E12.5 gonads cultured for 24h with a second VEGFR inhibitor, Axitinib. While endothelial cells and vasculature were unaffected by DMSO, culture with 100, 500 and 2500nM Axitinib disrupted vascular formation and abrogated pERK1/2 expression in endothelial cells, outcomes similar to those observed using VEGFRi (Additional file 16: Fig. S7B).

E12.5 gonads treated for 24h with DMSO or 500nM of VEGFRi were also analysed using wholemount IF to assess *Oct4*-eGFP, CD31 and pERK1/2 (Fig. 4B). As previously observed (Fig. 2B, Additional files 4–5: Movies S1-2), pERK1/2 was detected in *Oct4*-eGFP negative cells within the testis cords in DMSO controls, consistent with pERK1/2 expression in Sertoli cells (Fig. 4B, Additional files 17–18: Movies S9-10). In addition, pERK1/2 was detected in CD31 positive endothelial cells throughout the testis, particularly within the coelomic epithelium, between the testis cords and in the mesonephros (Fig. 4B, Additional files 17–18: Movies S9-10). In VEGFRi treated testes, pERK1/2 was not detected in the majority of endothelial cells outside the testis cords but was detected in *Oct4*-eGFP negative cells within the testis cords, consistent with maintained pERK1/2 in Sertoli cells (Fig. 4B, Additional files 19–20: Movies S11-12). pERK1/2 was also detected in cells at the epithelial surface of the gonad and in the mesonephros, however, these cells were CD31 negative indicating that they were not endothelial cells (Fig. 4B, Additional files 19–20: Movies S11-12). Together, as VEGFRi blocked pERK1/2 in endothelial cells and disrupted organisation of these cells into blood vessels, these data suggest that VEGF signalling promotes MEK1/2 signalling in CD31 expressing endothelial cells and their organisation into testicular vasculature. In contrast, given that pERK1/2 was still detected in Sertoli cells of VEGFRi treated testes, activation of pERK1/2 in Sertoli cells appears to be independent of VEGF signalling.

### SOX7/17 positive endothelial cells depend on VEGF signalling in the developing testis

**Fig. 5:**
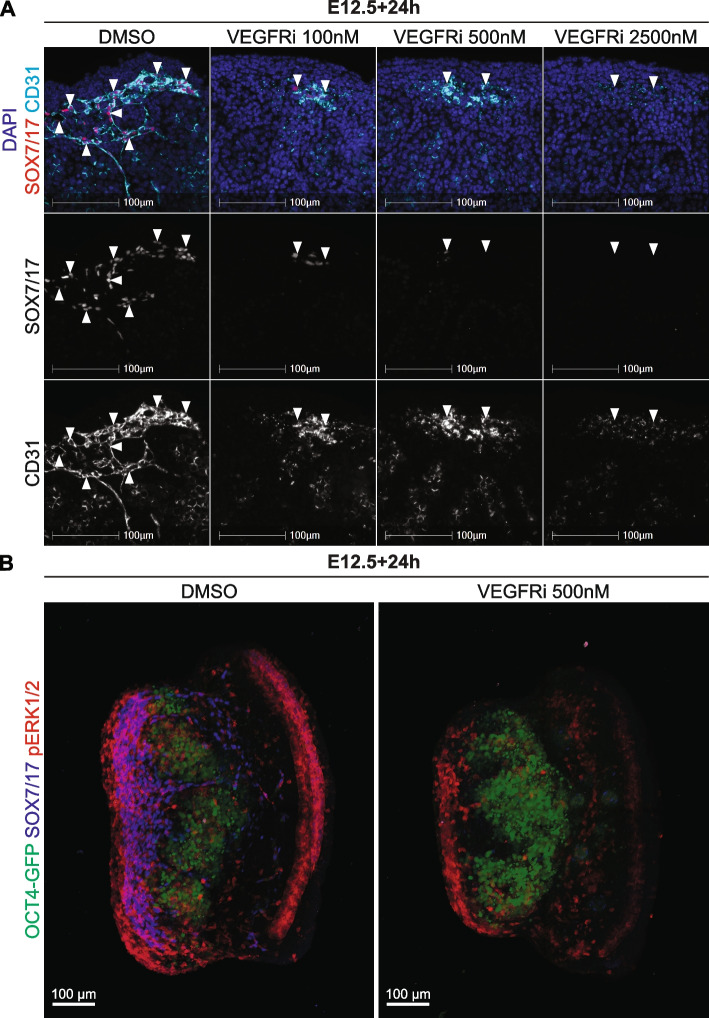
SOX7/17 positive endothelial cells depend on VEGFR signalling in the developing testis. Section and wholemount immunofluorescent imaging of E12.5 testes cultured for 24h with DMSO or 100, 500 or 2500 nM of VEGFRi. **A** IF in sections of cultured testes stained with DAPI (blue), SOX7/17 (red) and CD31 (endothelial cells and germ cells; cyan). **B** Three-dimensional stacked Z-series images of cultured testes showing *Oct4*-eGFP (germ cells; green), SOX7/17 (blue) and pERK1/2 (red). The DMSO and VEGFRi images should be viewed in conjunction with Movies S13-16 which reveal the 3D organisation of the cells in the tissue. Scale bar represents 100 μm. Arrows indicate endothelial cells. Biological replicates; *n* = 3–4 per treatment

We also assessed the expression of SOX7/SOX17 in VEGFRi testes to determine if endothelial cells expressing these key angiogenic transcription factors also depend on VEGF signalling. In DMSO controls treated for 24h, SOX7/17 was detected in CD31 expressing endothelial cells in the coelomic vessel (Fig. 5A, Additional file 21: S8A), as previously observed in DMSO controls for the MEKi experiments (Fig. 3A, Additional file 11: Fig S6A). Vasculature was substantially disrupted in testes treated with 100nm, 500nm or 2500nM VEGFRi for 24h, with fewer CD31 positive endothelial cells detected and those that were detected were not organised into recognisable blood vessels (Fig. 5A, Additional file 21: Fig. S8A). Moreover, SOX7/17 was undetected or almost undetectable in the remaining CD31 positive endothelial cells in 100nM VEGFRi treated samples and was undetected in 500 and 2500nM VEGFRi treated samples (Fig. 5A, Additional file 21: Fig. S8A). Moreover, CD31 positive endothelial cells were also detected in the mesonephros of VEGFRi treated gonads (Additional file 21: Fig. S8B), an outcome similar to that observed in MEKi treated gonads (Additional file 11: Fig. S6A-B). However, SOX7/17 was not detected or was substantially reduced in CD31 positive cells located in the mesonephros of VEGFRi treated gonads (Additional file 21: Fig. S8B). The observations in VEGFRi treated gonads were confirmed with 100-2500nM Axitinib, which eliminated SOX7/17 in CD31 positive endothelial cells and disrupted vasculature formation (Additional file 21: Fig. S8C). Together, as some CD31 cells remained in VEGFRi treated samples but SOX7/17 was lost, these data suggest that SOX7/17 expression depends on VEGF signalling in endothelial cells. Consistent with this and loss of VE-cadherin in gonads of samples treated with 500nM VEGFRi (Additional file 8: Fig S3), no CD31-pERK1/2 or CD31-SOX7/17 positive endothelial cells were detected in the gonad in samples treated for 72h with 500nM VEGFRi, despite robust detection of CD31 and pERK1/2 and CD31 and SOX7/17 in endothelial cells of control samples (Additional file 22: Fig. S9).

Wholemount IF analysis of *Oct4*-eGFP, SOX7/17 and pERK1/2 in E12.5 testes cultured with DMSO or VEGFRi for 24h produced similar results. In DMSO control testes, pERK1/2, SOX7/17 double positive cells were detected in the coelomic vessel, between the testis cords and in the mesonephros (Fig. 5B, Additional Files 23–24: Movies S13-14). In contrast, very few SOX7/17 positive cells were detected in the coelomic vessel, between the testis cords or within the mesonephros in VEGFRi treated testes, and the intensity of remaining SOX7/17 positive cells was substantially reduced compared to DMSO controls (Fig. 5B, Additional files 25–26: Movies S15-16). Moreover, the few SOX7/17 cells that were observed were negative for pERK1/2. Together, these data suggest that VEGF is required to mediate MEK1/2 signalling and ensure the presence of SOX7/17 expressing endothelial cells in the developing testis.

### VEGF and MEK1/2 signalling are required for endothelial cell survival

**Fig. 6:**
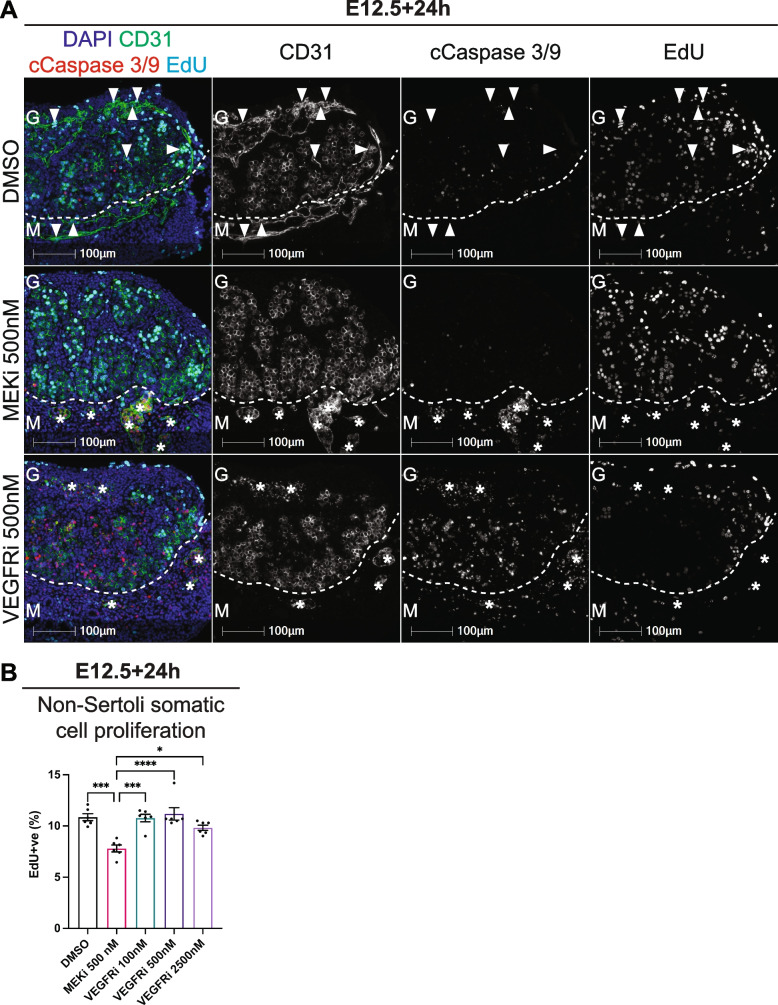
VEGF and MEK1/2 signalling is required for endothelial cell survival. E12.5 testes cultured for 24h with DMSO, 500nM of MEKi or 100, 500 or 2500nM of VEGFRi and analysed with immunofluorescence or flow cytometry. **A** IF in sections of cultured testes stained with DAPI (blue), CD31 (endothelial cells and germ cells; green), cleaved Caspase 3/9 (cCaspase 3/9; red) and EdU (cell proliferation; cyan). Key: G = gonad, M = mesonephros. Arrows indicate cCaspase 3/9 negative and EdU positive endothelial cells, asterisks indicate cCaspase 3/9 positive and EdU negative endothelial cells. **B** Flow cytometric analysis of non-Sertoli somatic cells with cell proliferation based on EdU incorporation. Scale bar represents 100 μm. Biological replicates; in A; *n* = 4 per treatment, in B; *n* = 6 testes per treatment. In B; ordinary one-way ANOVA with Tukey’s multiple comparisons. Data represents mean ± SEM. Only significance between treatments and DMSO or 500 nM MEKi are shown, *P < 0.05, ***P < 0.001, ****P < 0.0001

Given that there were no or very few CD31 positive endothelial cells detected in MEKi treated or VEGFRi treated testes respectively, compared to DMSO controls, we used IF to assess the cell death markers, cleaved Caspase3/9 (cCaspase3/9) together with 5-ethynyl-2’-deoxyuridine (EdU) incorporation to measure cell proliferation, and CD31 to identify endothelial cells. Flow cytometry was not used as we could not develop an appropriate assay using CD31. As previously demonstrated, CD31 positive endothelial cells were detected in an organised vasculature pattern between testis cords in the celomic vessel and in the gonad-mesonephric border in DMSO controls (Figs. 2, 3, 4 and 5). These cells did not express cCaspase3/9 and many but not all CD31 positive endothelial cells were proliferative as indicated by their incorporation of EdU (Fig. 6A, Additional file 27: Fig. S10A). In contrast, MEKi resulted in the absence of CD31 positive endothelial cells in the testis. However, some endothelial cells remained in the mesonephros but were not EdU positive and expressed cCaspase3/9, indicating that these cells had exited the cell cycle and were marked for death (Fig. 6A, Additional file 27: Fig. S10A). In addition, although endothelial cells were detected in the testes of VEGFRi treated samples, they were poorly organised and there appeared to be fewer of them compared to DMSO controls (Fig. 6A, Additional file 27: Fig. S10A). Furthermore, the endothelial cells detected in samples treated with 500nM VEGFRi were not proliferative and commonly expressed cCaspase3/9, indicating VEGFRi induced endothelial cell death in these samples.

In addition to endothelial cells detected in the DMSO controls, CD31 negative surface epithelial cells and other somatic cells within the testis incorporated EdU, indicating that these cells were also proliferative (Fig. 6A, Additional file 27: Fig. S10A). Moreover, surface epithelial cells detected in MEKi treated testes were EdU positive and cCaspase3/9 negative, indicating that survival of these cells does not depend on MEK1/2 signalling (Fig. 6A, Additional file 27: Fig. S10A). Additionally, some surface epithelial cells were also EdU positive in VEGFRi treated testes (Fig. 6A, Additional file 27: Fig. S10A). To confirm the IF data we used flow cytometry to quantify proliferation in VEGFRi- and MEKi-treated gonads. While we could not directly measure endothelial cell proliferation, staining of the Sertoli cells and germ cells using SOX9 and MVH (mouse vasa homolog) antibodies allowed examination of the double negative non-Sertoli cell population, which includes endothelial cells, interstitial cells and fetal Leydig cells. Following 24h of culture, MEKi resulted in a small but significant reduction in proliferation of non-Sertoli somatic cells compared to DMSO controls (*P* < 0.0001; Fig. 6B). In contrast, proliferation of non-Sertoli somatic cells was not affected by 100, 500 or 2500nM VEGFRi or 100 or 500nM Axitinib compared to DMSO controls (Fig. 6B, Additional file 27: Fig. S10B). At 2500nM Axitinib resulted in a significant reduction in non-Sertoli somatic cell proliferation compared to DMSO controls (*P* < 0.0001; Additional file 27: Fig. S10B), although significant accumulation at G2/M indicated that this high dose of Axitinib may induce cell death. Together, these data indicate that endothelial cells rely on VEGF and MEK1/2 signalling for cell survival and thus the maintenance of vasculature within the testis.

### Sertoli cell localisation depends on MEK1/2 but not VEGF signalling

**Fig. 7:**
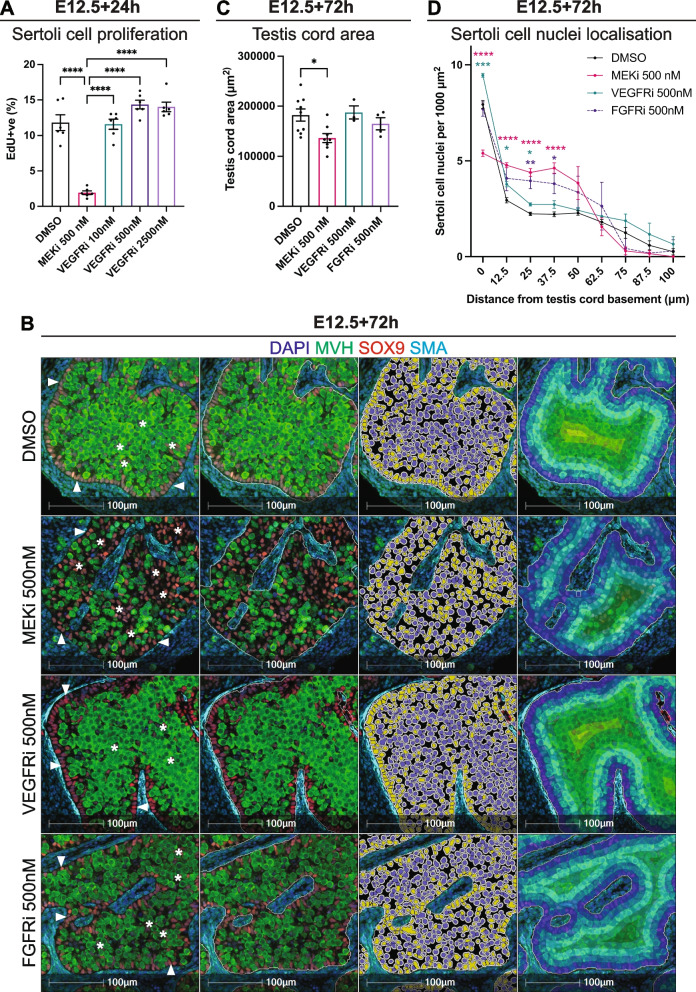
MEK1/2 signalling inhibition disrupts Sertoli cell proliferation and Sertoli cell localisation to the testis basement membrane. E12.5 testes cultured for 24 or 72h with DMSO, 500nM of MEKi, 100nM, 500nM or 2500nM of VEGFRi or 500nM of FGFRi and analysed with flow cytometry or immunofluorescence. **A** Flow cytometric analysis of Sertoli cells with cell proliferation based on EdU incorporation. **B** HALO AI analysis of Sertoli cell organisation in E12.5 testes cultured with DMSO, MEKi, VEGFRi or FGFRi. The first column of images shows representative staining of DAPI (blue), MVH (germ cells; green), SOX9 (Sertoli cells; red) and SMA (peritubular myoid cells; cyan). The second column of images demonstrates the region of interest (ROI) identified with HALO AI. The third column of images shows Sertoli cells selected based on SOX9 staining intensity. The last column shows the layers of Sertoli cells analysed using infiltration analysis. Arrows highlight Sertoli cells localised to the testis cord basement membrane and asterisks highlight Sertoli cells localised within the testis cords. Scale bar represents 500 μm (whole view images) or 100 μm (digital zoom images). **C** Testis cord area based on HALO AI image quantification. **D** Sertoli cell nuclei localisation relative to distance away from the testis cord basement membrane determined by HALO AI infiltration image analysis shown in B. Each bin represents 12.49 μm. Number of Sertoli cell nuclei presented as Sertoli cell nuclei/1000μm^2^. Biological replicates: In A; *n* = 6 per treatment, in B-D; *n* = 3–9 per treatment; 3 sections/gonad analysed. In A-B, statistics analysed using one-way ANOVA with Tukey’s multiple comparisons. In C, repeated measures two-way ANOVA with Geisser-Greenhouse correction and Tukey’s multiple comparisons. Data represents mean ± SEM. Significance between DMSO and treatments; * < 0.05, **P < 0.01, ***P < 0.001, ****P < 0.0001. For D; significance between DMSO and MEKi is represented by pink asterisks, significance between DMSO and VEGFRi is represented by teal asterisks, and significance between DMSO and FGFRi is represented by purple asterisks

We previously found that culturing E12.5 testes for 72h with MEKi resulted in significantly reduced Sertoli cell proliferation [13]. Consistent with this, flow cytometry confirmed that Sertoli cell proliferation was reduced in E12.5 testes treated for 24h with MEKi compared to DMSO controls (*P* < 0.0001; Fig. 7A). However, treatment with 100, 500 and 2500nM VEGFRi did not affect Sertoli cell proliferation (Fig. 7A), an outcome confirmed using 100, 500 and 2500nM Axitinib (Additional file 28: Fig. S11A). This is concordant with maintained pERK1/2 in Sertoli cells of VEGFRi-treated testes (Fig. 4, Additional file 16: Fig. S7).

To visualise the impacts of MEKi and VEGFRi on testis cord morphology and Sertoli cell development, we performed immunofluorescence (IF) analysis using antibodies against MVH and SOX9 to identify germ and Sertoli cells, respectively, while SMA (smooth muscle actin) expression in peritubular myoid cells was used to delineate testis cords. In DMSO controls, Sertoli cells were localised to the basement membrane of the testis cords (Fig. 7B, Additional file 28: Fig S11B). Similarly, in VEGFRi-treated samples, SOX9 positive Sertoli cells were also localised to the basement membrane of the testis cords after 72h of culture (Fig. 7B, Additional file 28: Fig. S11). However, in MEKi treated testes, while some Sertoli cells were localised to the testis cord basement membrane, many were distributed amongst germ cells throughout the testis cords (Fig. 7B, Additional file 28: Fig S11).

Using HALO AI (artificial intelligence) image analysis, the boundaries of the testis cords were identified, and the testis cord area measured. Although the area of the testis cords in VEGFRi-treated gonads was similar to DMSO controls, the testis cord area was significantly reduced in MEKi-treated samples (*P* < 0.05; Fig. 7C). To extend this analysis, we used HALO AI infiltration analysis to determine if Sertoli cells properly localised to the testis cord basement. This demonstrated a significant difference in the organisation of Sertoli cells within the testis cords of DMSO controls and gonads treated with MEKi. Less Sertoli cell nuclei were localised 0–12.49um from the basement membrane in MEKi-treated samples and more were detected in the cord interior within the bands 12.5–24.99, 25–37.49 and 37.5–49.99um from the basement (*P* < 0.0001, Fig. 7D). In contrast, slightly more Sertoli cell nuclei lined the testis cord basement membrane in VEGFRi-treated samples, than DMSO controls (Fig. 7D). Together, these data indicated that while MEK1/2 inhibition in E12.5 testes disrupted Sertoli cell localisation to the testis cord basement membrane, VEGFR inhibition did not.

Another ligand that may affect Sertoli cell localisation is FGF9, as FGF9-mediated signalling is essential for testis development and Sertoli cell proliferation [6–13]. To determine if reduced FGF signalling affected Sertoli cell nuclei localisation to the basement membrane we assessed testis cord area and applied infiltration analysis to E12.5 testes cultured with 500nM BGJ398 (FGFRi, Fig. 7B). We previously established that FGFRi at this concentration is sufficient to significantly reduce Sertoli cell proliferation in cultured E12.5 XY gonads and to completely abrogate FGF9 driven proliferation of XX supporting cells [13]. FGFRi did not significantly affect testis cord area (Fig. 7C). Moreover, infiltration analysis revealed that Sertoli cells in FGFRi-treated gonads occupied a level or organisation that was intermediate between MEKi-treated and DMSO controls gonads, demonstrating that inhibiting FGF signalling resulted in a more moderate effect on Sertoli cell localisation (Fig. 7D). While similar numbers of Sertoli cell nuclei were localised to the basement membrane (0–12.5um away from the basement), significantly more Sertoli cell nuclei remained in the 12.5–24.99 and 25–37.5um bands in FGFRi-treated samples than in DMSO controls (Fig. 7D). Although FGF-MEK1/2 signalling is rapid, this intermediate effect may be due to some initial FGF9 signalling in E12.5 gonads. However, this could not be tested as loss of FGF9 leads to testis to ovarian sex reversal, a major confounding factor for testis cord formation. Nonetheless, together our data indicate that FGF and MEK1/2 signalling are essential for proper localisation of Sertoli cells to the testis cord basement membrane during testis development.

## Discussion

We have identified novel and essential roles for MEK1/2 signalling in endothelial cell survival, fetal testis vascularisation and Sertoli cell development. Our RNA sequencing analysis revealed that MEK1/2 inhibition resulted in striking dysregulation of the transcriptional network associated with vasculogenesis and angiogenesis. Markers of endothelial cell development were reduced, and IPA strongly predicted that these transcriptional changes occurred downstream of VEGFA/VEGF signalling. Consistent with this, we demonstrated that VEGFR and MEK1/2 signalling are required for organisation of SOX7 and SOX17 expressing endothelial cells into vasculature and the survival of these cells in the developing testis. Finally, our data show that VEGF signalling is dispensable for Sertoli cell proliferation and localisation to the testis cord basement membrane. However, both FGF and MEK1/2 signalling are required for Sertoli cell proliferation, localisation and organisation within the testis cords. Together, this study reveals important roles for MEK1/2 in testicular vasculogenesis and testis cord organisation. Our data support a model in which VEGF signalling activates MEK1/2 to promote expression of the key angiogenic transcription factors *Sox7* and *Sox17* and drive development, organisation and survival of endothelial cells and vascularisation of the testis.

MEK1/2 inhibition disrupted 116 and 114 genes after 24 or 72h of culture, respectively. Although only 49/116 of the 24h DEGs were also dysregulated at 72h, IPA identified that similar biological functions were affected by 24 or 72h of MEK1/2 inhibition, including processes which rely on endothelial cell development such as angiogenesis, development of vasculature, and vasculogenesis. Furthermore, MEK1/2 inhibition resulted in lower expression of 40 genes identified as endothelial cell specific in an earlier study [44]. Although our study is limited in that we cannot determine whether the impacts of MEK1/2 signalling were direct or indirect, our data strongly indicate that testicular endothelial cells depend on MEK1/2 for their differentiation and survival. While vasculature development is initiated between E11.5 and E12.5 in the developing testis [4, 14] and we inhibited MEK1/2 signalling from E12.5, our data demonstrate that endothelial cells require MEK1/2 signalling to regulate the patterning of vasculature in the testis. The specific mechanisms involved in endothelial cell survival and vascular organisation are yet to be fully delineated, however, our data strongly suggest that the key vascular transcription factors SOX7/17 are involved.

IPA analysis predicted that 24h and 72 MEKi DEGs were downstream of VEGFA/VEGF signalling. Consistent with this, we demonstrated that inhibition of VEGF receptors eliminated pERK1/2 in CD31 positive cells, demonstrating that MEK1/2-ERK1/2 signalling was lost. Moreover, endothelial cells were substantially reduced in the testis and the formation of the coelomic vessel and other blood vessels was prevented in the tissue. This aligns with the established role for VEGF in testicular vasculogenesis [15, 16] and demonstrates that VEGF signals through MEK1/2-pERK1/2 to mediate testicular angiogenesis, an outcome that is consistent with the ability of VEGF to promote MEK1/2-ERK1/2 signalling in other biological contexts [33, 45–48].

**Fig. 8:**
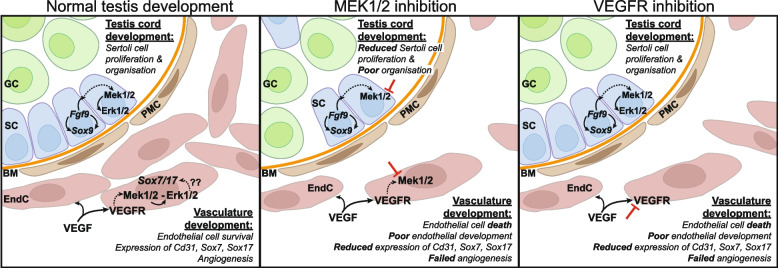
Proposed model for VEGF and MEK1/2 signalling in testis development. Between E11.5 and E12.5, endothelial cells migrate into and vascularise the testis, a process driven by VEGF signalling. VEGF signalling via VEGFR activates MEK1/2 and pERK1/2 to promote formation of endothelial cells, marked by SOX7/17 expression, which organise to form the testicular vasculature. In the developing testis, MEK1/2 inhibition disrupts endothelial cell development and endothelial cells are marked for apoptosis. Similarly, VEGFR inhibition compromises endothelial cell development and leads to endothelial cell apoptosis and failed vasculogenesis. MEK1/2-ERK1/2 signalling also regulates Sertoli cell proliferation and appropriate localisation to the testis cord basement membrane, a process that is likely mediated via FGF9 signalling. FGF9 production and testis development is supported via a positive feedback mechanism with SOX9 [9]. FGF or MEK1/2 inhibition reduces Sertoli cell proliferation and Sertoli cells fail to properly localise to the testis cord basement membrane. However, as VEGFR inhibition did not affect pERK1/2 expression in, or proliferation of Sertoli cells, we propose that VEGF activation of MEK1/2-ERK1/2 and *Sox7* and *Sox17* in endothelial cells is independent of MEK1/2-pERK1/2 signalling in Sertoli cells. GC: germ cell; SC: Sertoli cell; BM: basement mebrane; EndC: endothelial cell; PMC: peritubular myoid cell

Our RNA sequencing analysis provides substantive evidence that MEK1/2 signalling is required for the expression of at least 40 endothelial specific genes. In addition, we demonstrated that the angiogenesis genes, *Sox7* and *Sox17*, were downregulated and were also endothelial cell specific. Moreover, our IF analyses using MEK1/2 and VEGF inhibitors demonstrated that expression of CD31 and SOX7/17 depend on VEGF and MEK1/2 signalling and loss of VEGF or MEK1/2 signalling leads to endothelial cell death. Together, these observations indicate that endothelial cell survival depends on VEGF and MEK1/2 signalling, and loss of SOX7/17 can be explained by loss of endothelial cells. However, it also appears likely VEGF-MEK1/2 signalling is directly or indirectly required for SOX7/17 expression as SOX7/17 was substantially reduced in CD31 positive endothelial cells remaining in the mesonephros after 24h VEGR or MEK1/2 inhibition. In other contexts, the *SoxF* genes (*Sox7*, *Sox17* and *Sox18*) act as positive feedback regulators of VEGF signalling, a process that appears to be mediated via mTOR signalling [22]. Based on our data, we propose a model in which activation of VEGF signalling in testicular endothelial cells promotes MEK1/2-ERK1/2 signalling to ultimately activate *Sox7* and *Sox17* transcription, thereby driving angiogenesis. This may activate a range of downstream genes, such as the chemokine receptor, *Cxcr4*, and tight junction genes, *Gja4, Gja5* and *Cldn5*, which are required for blood vessel formation, and were reduced by MEK1/2 inhibition in this study (Fig. 8).

VEGF signalling is well established as a key pathway promoting fetal testis vasculature [15, 16, 49]. A previous study demonstrated that when VEGF signalling was inhibited from E11.5, endothelial cells were depleted, vasculature failed to be established within the gonad [15], and testis cords did not develop [15]. Similarly, inhibition of VEGF signalling from E12.5 disrupted vasculature, but did not affect testis cord development [16]. In this study, VEGF inhibition profoundly disrupted vasculature, but testis cord development appeared normal, an outcome consistent with Kumar and DeFalco [16]. Together these studies highlight an early temporal window during which VEGF signalling is important for both testis cord formation and vasculature patterning, and a slightly later window during which VEGF signalling is only required for vasculature patterning and maintenance. This likely reflects an early role of endothelial cells that migrate into the gonad at E11.5 in partitioning the Sertoli and germ cells into clusters that form cords, and a later role of endothelial cell differentiation and their assembly into the coelomic vessel and other testicular vasculature [4, 14].

MEK1/2 inhibition for 24 or 72h in E12.5 testes also resulted in reduced Sertoli cell proliferation and a failure of Sertoli cells to localise to the testis cord basement membrane. Despite the impact of MEK1/2 inhibition on proliferation and localisation, only five Sertoli cell specific genes (*Arhgdig*, *Etd*, *Gatm*, *Islr2* and *Rhox8/Tox*) were identified as dysregulated after 24h of culture. While the number of Sertoli cell expressed genes was low, genes that were dysregulated but not expressed specifically in Sertoli cells may also contribute to this phenotype. An additional explanation for the low number of dysregulated Sertoli cell genes may be that we inhibited MEK1/2 after Sertoli cell specification and early differentiation. Of these five dysregulated Sertoli cell specific genes, only *Etd* and *Rhox8/Tox* have been associated with testis development: *Etd* was downregulated following the loss of *Dmrt1* in Sertoli cells [50], while *Rhox8/Tox* has been demonstrated to be expressed in somatic cells of the fetal testis [51]. Furthermore, IPA did not identify any Sertoli cell specific biological functions. Consistent with this outcome, we previously demonstrated that inhibition of MEK1/2 in E12.5 testes does not cause loss of Sertoli cells or somatic cell sex reversal, eliminating sex-reversal as a confounding factor for the current study. Although Sertoli cell specification was not disrupted by MEK1/2 inhibition, Sertoli cell proliferation and localisation to the testis cord basement membrane was compromised, indicating a novel role for MEK1/2 signalling in Sertoli cell development and testis cord organisation.

While pERK1/2 was detected in both endothelial and Sertoli cells during normal development of E12.5, E14.5 and E15.5 testes, pERK1/2 was not detected in either endothelial cells or Sertoli cells following MEK1/2 inhibition. In contrast, while pERK1/2 was blocked by VEGFR inhibition in endothelial cells, VEGFR inhibition did not reduce pERK1/2 in Sertoli cells. In addition, while MEK1/2 inhibition reduced Sertoli cell proliferation, VEGFR inhibition did not. Together these findings indicate that in Sertoli cells the impacts of MEK1/2 signalling inhibition are independent of VEGF signalling and that another ligand is responsible for activating MEK1/2 signalling to promote Sertoli cell proliferation and organisation at the testis cord basement membrane. Our data indicate that FGF signalling at least contributes to this process. Although the impact of FGFR inhibition was less than that of MEK1/2 inhibition, it is well established that FGFs commonly activate MEK1/2-ERK1/2 signalling [52–55], and FGF9 has been demonstrated to promote and sustain pERK1/2 during murine mammary fibroblast proliferation [56]. Moreover expression of FGF9 from E11.5 is essential for mouse testis development and for proliferation and organisation of Sertoli cells in testis cords [6–13]. Our data are therefore consistent with a primary role for FGF9 in signalling via MEK1/2 to drive Sertoli cell proliferation and organisation to the testis cord basement membrane in developing testes [7].

## Conclusions

Together, our study reveals a novel pathway through which VEGF activates MEK1/2 signalling and SOX7/17, and is required for endothelial cell development and vasculogenesis in the developing testis. Our data also highlight a novel VEGF independent role for MEK1/2 signalling to promote Sertoli cell proliferation and organisation, likely to be mediated by FGF9. Further understanding of the molecular regulation of testicular vasculogenesis is likely to reveal how disruptions in this important vascular system may alter systemic distribution of physiologically critical factors such as testicular hormones, and how poor testicular blood circulation may compromise male reproductive outcomes such as endocrine health, testicular thermoregulation, and fertility.

## Methods

### Mouse strains, animal housing, breeding and ethics

Mice were housed at Monash Medical Centre Animal Facility with controlled temperature and humidity, a 12h light–dark cycle and food and water available ad libitum. Mouse embryos were obtained from inbred 129T2svJ *Oct4*-eGFP males crossed with Swiss females. Females were checked daily for vaginal plugs, with detection of a plug noted as E0.5. Animal work was undertaken in accordance with Monash Medical Centre Animal Facility Animal Ethics Committee approval.

### Organ culture

E12.5-E15.5 embryos were sexed visually by the presence (male) or absence (female) of testis cords in the gonad. Either hanging drop or membrane-based cultures were used to grow samples in an ex vivo setting. Regardless of whether hanging drop or membrane cultures were used, all E12.5 gonad-mesonephros samples were cultured in organ culture media (15mM Hepes, 0.1mM non-essential amino acids, 1mg/mL N-acetylcysteine, 1X penicillin/streptomycin, 55μM beta-mercaptoethanol and 10% fetal calf serum in DMEM/F12 with Glutamax) containing DMSO (vehicle control), 500nM of MEKi (PD0325901; SelleckChem, S1036), 100, 500 or 2500nM of VEGFRi (cabozantinib; MedChem Express, HY-13016) or Axitinib (second VEGFR inhibitor; MedChem Express, HY-10065), or 500nM of FGFRi (BGJ398; SelleckChem, HY-13241). These inhibitors were selected based on its high-specificity, potency (IC50 – MEK: 0.33 nM, VEGFRi: 0.035-12nM VEGFR1,2,3, Axitinib: 0.1–0.2nM VEGFR1,2,3; FGFRi: FGFR1–3: 0.9–1.4 nM, FGFR4: 60 nM) and advancement in clinical trials (Phase II, NCT03962543, Phase IV, NCT01896479, Phase III, NCT00678392, Phase II, NCT02150967). Gonad-mesonephric complexes were randomly allocated to each culture treatment condition and cultured for 24 or 72h in 5% CO_2_ at 37°C, with media refreshed daily. Gonad-mesonephric complexes were cultured for 24 or 72h on 30mm Millicell Biopore membranes with 0.4μm pores (Merck Millipore; PICM03050) in 6-well plates, with each well containing 1400μL media and PBS was placed in between the wells to maintain humidity. To facilitate analysis of cell proliferation, EdU was added to each sample for the final two hours of culture at a final concentration of 20μM. For gonads used for wholemount IF, gonad-mesonephric complexes cultured for 24h in organ culture media using 30 μL hanging drops in the lid of a petri dish, with PBS placed in the base dish to maintain humidity. After culture, gonads were processed for flow cytometry, section and wholemount IF or FACS.

### Flow cytometry

Gonad collection, dissociation, fixation, staining and flow cytometry were performed essentially as described previously [57], using antibodies specific for MVH and SOX9. Mesonephros samples were used as germ cell negative controls to set gates for MVH and E12.5 female gonads were used as a negative control to set gates for SOX9 (Additional file 29: Fig. S12). Primary antibodies used include MVH and SOX9 (Table 1). Secondary antibodies used include Alexa Fluor Donkey anti Goat 488 (Thermo-Fisher, A11055) and Biotin-linked Donkey anti Rabbit (Thermo-Fisher, A16027). Cell cycle analysis was performed as previously described [57], with germ cells, Sertoli cells and non-Sertoli somatic cells identified by expression of MVH, SOX9 or neither, respectively. Cells were stained with 20μg/mL of propidium iodide, enabling quantitation of cellular DNA content. Proliferation was measured by gating EdU positive cells to identify cells in S-phase, while cells in G0/G1 or G2/M were respectively identified by DNA content estimated as 2n or 4n in the EdU negative population compared to DMSO controls. All flow cytometry was performed on a BD LSR Fortessa analyser (BD, Biosciences).

**Table 1:** Antibodies for flow cytometric and immunofluorescence (IF) experiments

Protein	Source and catalogue number	Species	Dilution
Mouse Vasa Homologue (MVH)	R&D Systems, AF2030	Goat	F: 1/100 SIF: 1/400
CD31	R&D Systems, AF3628	Goat	SIF: 1/100 WIF: 1/100
SOX7	R&D Systems, AF2766	Goat	SIF: 1/800 WIF: 1/800
SOX17	R&D Systems, AF1924	Goat	SIF: 1/800 WIF: 1/800
SOX9	Sigma-Aldrich, AB5535	Rabbit	F: 1/200 SIF: 1/1000
SOX9	R&D Systems, AF3075	Goat	SIF: 1/2000
CD31	Cell Signalling Technology, 77699S	Rabbit	SIF: 1/400
Phospho-ERK1/2 (pERK1/2)	Cell Signalling Technology, 4370S	Rabbit	SIF: 1/200 WIF: 1/200
Cleaved Caspase 3	Cell Signalling Technology, 9661S	Rabbit	SIF: 1/400
Cleaved Caspase 9	Cell Signalling Technology, 9509S	Rabbit	SIF: 1/1000
Smooth Muscle Actin (SMA)	Sigma-Aldrich, A2547	Mouse	SIF: 1/1000
VE-cadherin	R&D Systems, AF1002	Goat	SIF: 1/800

*F* Flow cytometry, *SIF* Section immunofluorescence, *WIF* Wholemount immunofluorescence

### Section immunofluorescence and imaging

Gonads were fixed in 4% paraformaldehyde (PFA) in PBS overnight at 4°C. Samples were washed three times in PBS before 70% ethanol processing and embedded in paraffin. 4μm sections were cut in a compound series over four slides with around 8–10 sections collected/slide, resulting in a section every 16um through the whole tissue for each gonad sample stained with each antibody combination. Sections were mounted on Superfrost Plus slides and dried at least overnight before antibody incubation. Antigen retrieval was conducted using Dako citrate buffer (pH 6.0) for 30 min at 98°C in a PT Link rinse station (Dako). Tissue sections were blocked in PBS containing 5% BSA (Merck, A9647) and 10% donkey serum (Sigma; D9663) for 1h at RT. Primary antibody (Table 1) diluted in PBS containing 1% BSA was left to incubate overnight at 4°C or for 2h room temperature (RT). Slides were incubated for 1h at RT in the dark in secondary antibody (Alexa Fluor, Thermo-Fisher, Donkey anti Goat 488 A11055; Donkey anti Rabbit 647 A31573 or Donkey anti Mouse 647 A31571; Donkey anti Mouse 555 A31570; Donkey anti Goat 555 A21432) diluted at 1/500 in PBS containing 1% BSA. Cell proliferation based on EdU incorporation was assessed using Click-iT® EdU Alexa Fluor® 647 Flow Cytometry Assay Kit (Thermo Fisher Scientific), with reagents and instructions adapted to suit IF staining, including a final concentration of 5μM Alexa Fluor 647 Azide, Triethylammonium Salt (Thermo Fisher Scientific) for staining. Slides were incubated in DAPI (Thermo-Fisher) diluted at 1/5000 in distilled H_2_O for 15 min at RT. To quench autofluorescence the slides were incubated in 0.3% Sudan Black (Sigma) diluted in 70% ethanol for 30 seconds. The Sudan Black was rinsed off with distilled H_2_O and mounted in Prolong Gold (Thermo Fisher Scientific). All slides were scanned on the Olympus VS120 slide scanner by the Monash Histology Platform. The same laser settings were used for each experimental run.

### Fluorescent activated cell sorting (FACS) of somatic cells

After organ culture, the mesonephros was dissected from the gonads. Six to 15 gonads were pooled for each sample and were dissociated in trypsin. Somatic cell populations were isolated as previously described [57, 58] using the BD FACSAria™ Fusion cell sorter. GFP negative somatic cell populations were isolated from E12.5 XY control gonads and E12.5 XY gonads cultured for 24 or 72h in DMSO or MEKi (*n* = 4 for each treatment/group). Somatic cells were defined as GFP negative, with dead propidium iodide positive somatic cells excluded.

### RNA-sequencing library construction and sequencing

RNA was isolated from 20–45 × 10^4^ FACS-sorted somatic cells using Macherey–Nagel NucleoSpin® RNA XS extraction kit (Scientifix, 740,902.50) following manufacturer instructions. RNA quantity and RNA integrity (RIN) were assessed using Qubit and Bioanalyzer (Agilent Technologies). Libraries were prepared with 30ng of RNA from somatic cells with RIN values greater than 7. The library was constructed by the MHTP Medical Genomics Facility as previously described [59]. Briefly, during initial poly(A) tail priming, an 8bp sample index along with a 10bp unique molecular identifier (UMI) was added. Samples were pooled and amplified with a template switching oligonucleotide. The Illumina P5 and P7 were added by PCR and Nextera transposase, respectively. The forward read (R1) utilised a custom primer to sequence into the index while the reverse read (R2) used a standard R2 primer to sequence the cDNA in the sense direction. I7 indexes were added to enable parsing of sample sets. Sequencing was performed on NextSeq2000 (Illumina) following Illumina protocol 1,000,000,109,376 v3.

### Data pre-processing

FASTQ files were demultiplexed and mapped using scPipe [60] and Rsubread [61] (Bioconductor) packages in R studio. Briefly, FASTQ files were reformatted with *sc_trim_barcode* to incorporate barcode information from read 1 into read 2 into the header. Reads were aligned to a reference mouse genome (GENECODE GRCm39) using Rsubread. Reads were assigned to annotated exons with sc*_exon_mapping*, data were demultiplexed using *sc_demultiplex* and a gene count matrix was generated with UMI deduplication using *sc_gene_counting*. Gene count matrices from each set were combined into a DGEList object for analysis.

### Downstream analysis of RNA-sequencing data

Differential gene expression was assessed using the limma [62], Glimma [63] and edgeR [64] Bioconductor packages following a previously established workflow [65]. Briefly, gene count data was loaded into R studio and genes were annotated with any duplicates removed. Raw counts were transformed into counts per million (CPM). Lowly expressed genes were removed using the *filterByExpr* function in edgeR, and gene expression distributions were normalised using trimmed mean of M-values (TMM) method [66]. Multidimensional scaling (MDS) plots were generated to visualise sample clustering. Heteroscedasticity was removed from the count data with *voomWithQualityWeights* [67]. Linear modelling and empirical Bayes moderations was used to test for differential expression. As there were many DEGs identified following empirical Bayes moderations, to identify DEGs of biological relevance, a log_2_fold-change cut-off was set at greater/less than 0.585 (equivalent to a FC of 1.5) using *treat* [68]. Genes were considered differentially expressed if they met the logFC cut-off and had an FDR adjusted *p*-value less than 0.05. IPA [90, 348, 151] was used to identify biological functions associated with DEGs identified in the somatic cell population for each treatment. Upstream analysis was also performed to identify potential upstream regulators of MEK1/2 signalling in the somatic cell population. Venn diagrams were generated using InteractiVenn [69]. All RNA sequencing data has been deposited in the Gene Expression Omnibus (GEO) and are publicly available via accession numbers GSE221453 and GSE221458.

### Wholemount immunofluorescence

Gonads were processed and stained as previously described [70]. Gonads were fixed in 4% PFA in PBS overnight at 4°C. All incubation periods were performed in the dark, unless otherwise stated. Prior to staining gonads incubated in 0.1% Triton X in PBS (PBTx) at 4°C for 1h. Non-specific binding was blocked using PBTx containing 10% heat inactivated donkey serum for at least 2 h at 4°C. Gonads were then resuspended in primary antibody (Table 1) diluted in PBTx containing 10% heat inactivated donkey serum and incubated for at least 48h at 4°C. Following incubation, the gonads were washed in PBTx overnight at 4°C, with the PBTx replaced three times. The gonads were then incubated secondary antibody (donkey anti rabbit 568 Thermo Fisher Scientific, A10042 and donkey anti goat 647 Thermo Fisher Scientific, A21447) diluted in PBTx containing 10% heat inactivated donkey serum for at least 48h at 4°C in the dark. Following incubation, the gonads were washed in PBTx overnight at 4°C, with the PBTx replaced three times. Samples were fixed in 4% PFA (%w/v) in PBS for 10 min at RT. The gonads were mounted in a 10 mm fluoro dish in 1.5% agarose gel dissolved in TAE buffer. Once the gel was set, PBS was added on top of the agarose gel to prevent the gel from drying out and to facilitate imaging. Gonads were imaged on an Olympus FV-MPERS multiphoton microscope using a 25 × objective lens using a step size of 5 μm. Images were processed on Imaris × 64 (9.2.1).

### Statistical analysis

Flow cytometric data was analysed with FlowJo (v10.8.2). Data represents 4–7 biological replicates (outlined in figure legends and depicted in graphs). Image analysis was conducted using HALO (Indica Labs). Data from at least three representative tissue sections from the centre of the gonad were analysed per biological replicate, with 6–7 biological replicates for each group. Statistical significance was determined with GraphPad Prism (v10.0.3) using unpaired t-tests or repeated measures two-way ANOVA with Geisser-Greenhouse correction with Tukey’s multiple comparisons, ordinary one-way ANOVA with Tukey’s multiple comparisons or non-parametric equivalent where appropriate. *P* values < 0.05 were considered significant. All error bars represent mean ± SEM. All experiments were replicated at least twice, with limited variation between experiments.

## Supplementary Information

Additional file 1: Tables S1.1—S1.4. Supplementary Table containing gene lists generated from RNA sequencing analyses performed in this study.

Additional file 2: Fig. S1. The downstream target of MEK1/2, pERK1/2 (phosphorylated ERK1/2) is strongly detected in endothelial cells and weakly detected in Sertoli cells in E12.5-E15.5 testes. Immunofluorescent imaging of testes collected from E12.5, E13.5, E14.5 and E15.5 embryos and stained with DAPI (blue), pERK1/2 (red) and CD31 (endothelial cells and germ cells; cyan). Arrows indicate pERK1/2 positive endothelial cells and asterisks indicate pERK1/2 positive Sertoli cells. Scale bar represents 500 μm in whole view images (first panel) or 100 μm in digital zoom images (right three panels). Biological replicates; *n*  = 4 testes per stage.

Additional file 3: Fig. S2. Immunofluorescent images of E12.5 testes cultured for 24 or 72 h with DMSO or 500 nM MEKi. A) Wide field images of sections from E12.5 testes cultured for 24 (i) or 72 h (ii) shown in Fig. 3. Images show DAPI (blue), pERK1/2 (red) and CD31 (endothelial cells and germ cells; cyan). B) IF images showing SOX9 and pERK1/2 double staining in testis sections of DMSO controls and MEKi-treated samples after 24 h of culture. DAPI (blue), pERK1/2 (green) and SOX9 (red). Arrows indicate pERK1/2 positive Sertoli cells. Scale bar represents 100 μm for 24 h samples or 500 μm for 72 h samples. Biological replicates; *n* = 4 testes per treatment.

Additional file 4: Movie S1. Movie of an E12.5 testis cultured with DMSO for 24h showing *Oct4*-eGFP (germ cells) in green, pERK1/2 in red and CD31 (endothelial cells and germ cells) in blue.

Additional file 5: Movie S2. Movie of an E12.5 testis cultured with DMSO for 24h showing *Oct4*-eGFP (germ cells) in green, pERK1/2 in red and CD31 (endothelial cells and germ cells) in blue.

Additional file 6: Movie S3. Movie of an E12.5 testis cultured with 500 nM MEKi for 24h showing *Oct4*-eGFP (germ cells) in green, pERK1/2 in red and CD31 (endothelial cells and germ cells) in blue.

Additional file 7: Movie S4. Movie of an E12.5 testis cultured with 500 nM MEKi for 24h showing *Oct4*-eGFP (germ cells) in green, pERK1/2 in red and CD31 (endothelial cells and germ cells) in blue.

Additional file 8: Fig. S3. Epithelial cell marker, VE-cadherin, is lost in gonads treated with MEK1/2 inhibitor for 24 h. Immunofluorescence images showing VE-cadherin and CD31 double staining in gonad sections of DMSO controls and 500 nM MEKi or 500 nM VEGFRi-treated samples after 24 h of culture. DAPI (blue), VE-cadherin (red) and CD31 (cyan). Scale bar represents 500 μm for wide field images or 100 μm in digital zoom images. Biological replicates; *n* = 4 testes per treatment. Key: G – gonad, M – mesonephros.

Additional file 9: Fig. S4. Angiogenesis markers, SOX7 and SOX17, are expressed in endothelial cells. Immunofluorescent images of testes collected from E12.5 and E15.5 embryos and stained with DAPI (blue), SOX7 (red; A) or SOX17 (red; B) or SOX7/17 (red; C) and CD31 (endothelial cells and germ cells; cyan). Scale bar represents 500 μm in whole view images (first panel) or 100 μm in digital zoom images (right three panels). Biological replicates; *n* = 3 testes per stage.

Additional file 10: Fig. S5. SOX7/17 is detected in endothelial cells in E12.5-E15.5 testes. Immunofluorescent imaging of testes collected from E12.5, E13.5, E14.5 and E15.5 embryos, stained with DAPI (blue), SOX7/17 (red) and CD31 (cyan). Arrows indicate SOX7/17 positive endothelial. Testes were obtained from embryos collected directly from pregnant females at E12.5, E13.5, E14.5 and E15.5. Scale bar represents 500 μm in whole view images (first panel) or 100 μm in digital zoom images (right three panels). Arrows indicate SOX7/17 positive endothelial cells. Biological replicates; *n* = 4 testes per stage.

Additional file 11: Fig. S6. Wide field images and higher power images of the gonad-mesonephric border of testis sections shown in Fig. 4. Immunofluorescent wide field images (A and C) or higher power images of the gonad-mesonephric border (B) of E12.5 testes cultured for 24 (A and B) or 72 h (C) with DMSO or 500 nM of MEKi stained with DAPI (blue), SOX7/17 (red) and CD31 (endothelial cells and germ cells; cyan). Scale bar represents 100 μm (A and B) or 500 μm (C). Biological replicates; *n* = 4 testes per treatment. Key: G = gonad, M = mesonephros.

Additional file 12: Movie S5. Movie of an E12.5 testises cultured with DMSO for 24h showing *Oct4*-eGFP (germ cells) in green, pERK1/2 in red and SOX7/17 in blue.

Additional file 13: Movie S6. Movie of an E12.5 testis cultured with DMSO for 24h showing *Oct4*-eGFP (germ cells) in green, pERK1/2 in red and SOX7/17 in blue.

Additional file 14: Movie S7. Movie of an E12.5 testis cultured with 500 nM MEKi for 24h showing *Oct4*-eGFP (germ cells) in green, pERK1/2 in red and SOX7/17 in blue.

Additional file 15: Movie 8. Movie of an E12.5 testis cultured with 500 nM MEKi for 24h showing *Oct4*-eGFP (germ cells) in green, pERK1/2 in red and SOX7/17 in blue.

Additional file 16: Fig. S7. VEGFR inhibition reduces CD31 and pERK1/2 in developing testes. A) Wide field immunofluorescent images of E12.5 testes cultured for 24 h with DMSO or 100, 500 or 2500 nM of VEGFRi stained with DAPI (blue), pERK1/2 (red) and CD31 (endothelial cells and germ cells; cyan). Scale bars represent 100 μm. B) Immunofluorescent images of E12.5 testes cultured for 24 h with DMSO or 100, 500 or 2500 nM of Axitinib (a second VEGFR inhibitor) stained with DAPI (blue), pERK1/2 (red) and CD31 (cyan). Scale bars represent 500 μm (top panel) or 100 μm (bottom three panels). Arrows indicate endothelial cells and asterisks indicate pERK1/2 positive Sertoli cells. Biological replicates; *n* = 4 testes per stage.

Additional file 17: Movie S9. Movie of an E12.5 testis cultured with DMSO for 24h showing *Oct4*-eGFP (germ cells) in green, pERK1/2 in red and CD31 (endothelial cells and germ cells) in blue.

Additional file 18: Movie S10. Movie of an E12.5 testis cultured with DMSO for 24h showing *Oct4*-eGFP (germ cells) in green, pERK1/2 in red and CD31 (endothelial cells and germ cells) in blue.

Additional file 19: Movie S11. Movie of an E12.5 testis cultured with 500 nM VEGFRi for 24h showing *Oct4*-eGFP (germ cells) in green, pERK1/2 in red and CD31 (endothelial cells and germ cells) in blue.

Additional file 20: Movie 12. Movie of an E12.5 testis cultured with 500 nM VEGFRi for 24h showing *Oct4*-eGFP (germ cells) in green, pERK1/2 in red and CD31 (endothelial cells and germ cells) in blue.

Additional file 21: Fig. S8. VEGFR inhibition reduces SOX7/17 in developing testes. A-B) Immunofluorescent wide field (A) or higher power images of the gonad-mesonephric border (B) of E12.5 testes cultured for 24 h with DMSO or 100, 500 or 2500 nM of VEGFRi stained with DAPI (blue), SOX7/17 (red) and CD31 (endothelial cells and germ cells; cyan). Scale bars represent 100 μm. C) Immunofluorescent imaging of E12.5 testes cultured for 24 h with DMSO or 100, 500 or 2500 nM of Axitinib (a second VEGFR inhibitor) stained with DAPI (blue), SOX7/17 (red) and CD31 (cyan). Scale bars represent 500 μm (top panel) or 100 μm (bottom three panels). Arrows indicate endothelial cells. Key: G = gonad, M = mesonephros. Biological replicates; *n* = 4.

Additional file 22. Fig. S9. VEGF inhibition prevents endothelial cell activation of pERK1/2 and SOX7/17 expression in the developing testes. Immunofluorescent images E12.5 testes cultured for with DMSO or 500 nM of VEGFRi for 72 h showing DAPI (blue), pERK1/2 (red; A), SOX7/17 (red; B) and CD31 (cyan). Scale bars represent 500 μm (whole view images) or 100 μm (digital zoom images).

Additional file 23: Movie S13. Movie of an E12.5 testis cultured with DMSO for 24h showing *Oct4*-eGFP (germ cells) in green, pERK1/2 in red and SOX7/17 in blue.

Additional file 24: Movie S14. Movie of an E12.5 testis cultured with DMSO for 24h showing *Oct4*-eGFP (germ cells) in green, pERK1/2 in red and SOX7/17 in blue.

Additional file 25: Movie S15. Movie of an E12.5 testis cultured with 500 nM VEGFRi for 24h showing *Oct4*-eGFP (germ cells) in green, pERK1/2 in red and SOX7/17 in blue.

Additional file 26: Movie 16. Movie of an E12.5 testis cultured with 500 nM VEGFRi for 24h showing *Oct4*-eGFP (germ cells) in green, pERK1/2 in red and SOX7/17 in blue.

Additional file 27: Fig. S10. Endothelial cells are lost in E12.5 testes treated with MEK1/2 or VEGFR inhibitor. A) Immunofluorescent images of E12.5 testes cultured for with DMSO or 500 nM of MEKi or VEGFRi for 24 h stained for DAPI (blue),  CD31 (green), cCaspase 3/9 (red) and EdU (cell proliferation; cyan). Scale bars represent 500 μm. B) Flow cytometric analysis of non-Sertoli somatic cell proliferation based on EdU incorporation in E12.5 testes cultured for 24 h with DMSO, 500 nM of MEKi or 100, 500, or 2500 nM of Axitinib. Biological replicates: In A; *n* = 4 per treatment, in B; *n* = 4–6. Statistics analysed using one-way ANOVA with Tukey’s multiple comparisons. Data represents mean ± SEM. Significance between DMSO and treatments; * < 0.05,**P < 0.01, ***P < 0.001, ****P < 0.0001.

Additional file 28: Fig. S11. MEK1/2 signalling inhibition disrupts Sertoli cell proliferation and Sertoli cell localisation to the testis basement membrane. A) Flow cytometric analysis of E12.5 testes cultured with DMSO or 100, 500 or 2500 nM of Axitinib showing Sertoli cell proliferation based on EdU incorporation. B) HALO AI analysis of Sertoli cell organisation in E12.5 testes cultured with DMSO or 500 nM ofMEKi, VEGFRi or FGFRi. The first column of images shows representative staining of DAPI (blue), MVH (germ cells; green), SOX9 (Sertoli cells; red) and SMA (peritubular myoid cells; cyan). The second column of images demonstrates the region of interest (ROI) identified with HALO AI. The third column of images shows Sertoli cells selected based on SOX9 staining intensity. The last column shows the layers of Sertoli cells analysed using infiltration analysis. Biological replicates: A; *n* = 4–6. B; *n* = 3–9 per treatment. Statistics analysed using one-way ANOVA with Tukey’s multiple comparisons. Data represents mean ± SEM. Significance between DMSO and treatments; * < 0.05,**P < 0.01, ***P < 0.001, ****P < 0.0001.

Additional file 29: Fig. S12. Gating strategy and controls for flow cytometric analysis. A = Area, W = Width. A) Single cells were identified based on propidium iodide (PI) width vs area plots. Somatic and germ cells were separated based on MVH expression with germ cells identified as MVH positive and somatic cells identified as MVH negative. The somatic cell population was separated into SOX9 expressing Sertoli cells and SOX9 negative non-Sertoli somatic cells. Cell cycle analysis was performed In the Sertoli cell and non-Sertoli somatic cell populations based on EdU incorporation to identify cells in S-phase, with EdU negative cells in G0/G1 and G2/M identified according to DNA content determined by PI staining intensity (cells in G0/G1 have low PI intensity / 2N DNA content while cells in G2/M have high PI intensity / 4N DNA content). B) Appropriate controls were used to set gating including mesonephric cells to identify MVH negative cells (i), ovarian cells to identify SOX9 negative cells (ii) and cells not treated with EdU were used to identify the EdU negative population (iii).

Additional file 30. Supporting data values.

## Data Availability

All data generated or analysed during this study are included in this published article, its supplementary information files and publicly available repositories. The RNA sequencing data have been deposited in the Gene Expression Omnibus (GEO) and are publicly available via accession numbers GSE221458 and GSE221453. GSE221458 [Blücher RO, Lim RS, Jarred EG, Ritchie ME and Western PS. FGF-independent MEK1/2 signalling is essential for male germline development in mice] (Somatic Cell) and GSE221453 [Blücher RO, Lim RS, Jarred EG, Ritchie ME, Western PS. FGF- independent MEK1/2 signalling is essential for male germline development in mice] (Germ Cell). As somatic and germ cell data were analysed together, both datasets are supplied here. Individual values for Main Figures and Supplementary data are shown in Additional file 30.

## Abbreviations

3β-HSD: 3β-Hydroxysteroid dehydrogenase
EdU: 5-Ethynyl-2’-deoxyuridine
AI: Artificial intelligence
cCaspase3/9: Cleaved Caspase3/9
CPM: Counts per million
DSD: Differences of sex development
DEG: Differentially expressed gene
E: Embryonic day
ERK: Extracellular signal-regulated kinases
FDR: False discovery rate
FGFRi: FGF receptor inhibitor/BGJ398
Fgf9: Fibroblast growth factor 9
FACS: Fluorescent activated cell sorting
FC: Fold-Change
GEO: Gene Expression Omnibus
IF: Immunofluorescence
IPA: Ingenuity Pathway Analysis
MEKi: MEK1/2 inhibitor/PD0325901/Mirdametinib
MAPK: Mitogen-activated protein kinase
MEK: Mitogen-activated protein kinase kinase
MVH: Mouse vasa homolog
MDS: Multidimensional scaling
PFA: Paraformaldehyde
pERK1/2: Phospho-ERK1/2
PDGF-AA: Platelet-derived growth factor AA
RIN: RNA integrity number
RT: Room temperature
Sry: Sex-determining region Y
SMA: Smooth muscle actin
Sox9: SRY box 9
Tie2: TEK receptor tyrosine kinase
TMM: Trimmed mean of M-values
UMI: Unique molecular identifier
VEGF: Vascular endothelial growth factor
Vegfr2: VEGF receptor 2
VEGFRi: VEGFR inhibitor/Cabozantinib
