# Supplementary material for: VEGF-dependent testicular vascularisation involves MEK1/2 signalling and the essential angiogenesis factors, SOX7 and SOX17
Source: BMC Biol. 2024 Oct 1;22:222. doi: 10.1186/s12915-024-02003-y (PMC11445939; doi:10.1186/s12915-024-02003-y)
Supplement: Supplementary file 2 — Additional file 2: Fig. S1. The downstream target of MEK1/2, pERK1/2 (phosphorylated ERK1/2) is strongly detected in endothelial cells and weakly detected in Sertoli cells in E12.5-E15.5 testes. Immunofluorescent imaging of testes collected from E12.5, E13.5, E14.5 and E15.5 embryos and stained with DAPI (blue), pERK1/2 (red) and CD31 (endothelial cells and germ cells; cyan). Arrows indicate pERK1/2 positive endothelial cells and asterisks indicate pERK1/2 positive Sertoli cells. Scale bar represents 500 μm in whole view images (first panel) or 100 μm in digital zoom images (right three panels). Biological replicates; n = 4 testes per stage. [file 12915_2024_2003_MOESM2_ESM.pdf]

Figure S1

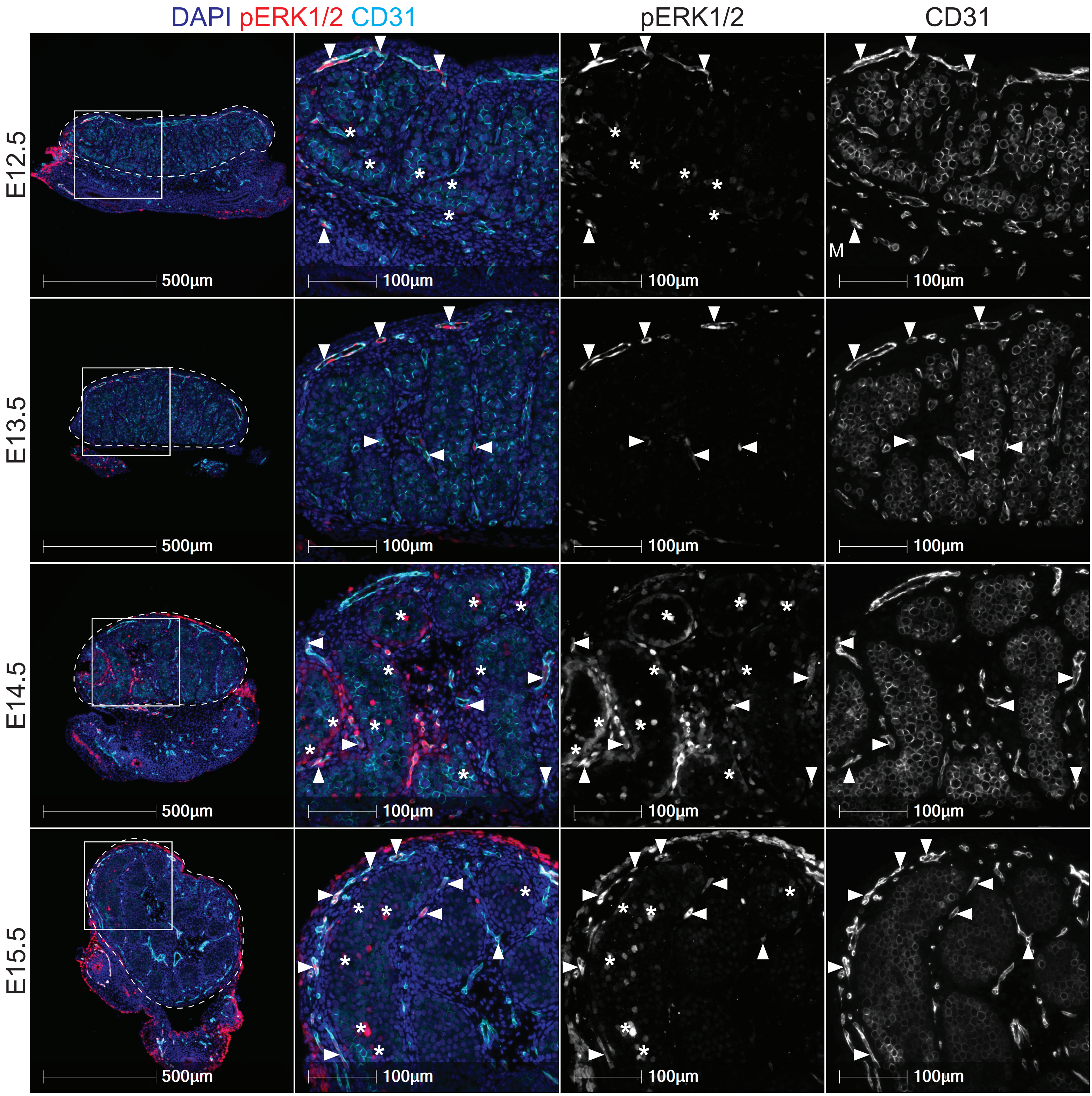
DAPI pERK1/2 CD31
pERK1/2
CD31
E12.5
E13.5
E14.5
E15.5
M
500µm
100µm

Additional file 2: Fig. S1. The downstream target of MEK1/2, pERK1/2 (phosphorylated ERK1/2) is strongly detected in endothelial cells and weakly detected in Sertoli cells in E12.5-E15.5 testes. Immunofluorescent imaging of testes collected from E12.5, E13.5, E14.5 and E15.5 embryos and stained with DAPI (blue), pERK1/2 (red) and CD31 (endothelial cells and germ cells; cyan). Arrows indicate pERK1/2 positive endothelial cells and asterisks indicate pERK1/2 positive Sertoli cells. Scale bar represents 500 μm in whole view images (first panel) or 100 μm in digital zoom images (right three panels). Biological replicates; n = 4 testes per stage.
