# Supplementary material for: VEGF-dependent testicular vascularisation involves MEK1/2 signalling and the essential angiogenesis factors, SOX7 and SOX17
Source: BMC Biol. 2024 Oct 1;22:222. doi: 10.1186/s12915-024-02003-y (PMC11445939; doi:10.1186/s12915-024-02003-y)
Supplement: Supplementary file 3 — Additional file 3: Fig. S2. Immunofluorescent images of E12.5 testes cultured for 24 or 72 h with DMSO or 500 nM MEKi. A) Wide field images of sections from E12.5 testes cultured for 24 (i) or 72 h (ii) shown in Fig. 3. Images show DAPI (blue), pERK1/2 (red) and CD31 (endothelial cells and germ cells; cyan). B) IF images showing SOX9 and pERK1/2 double staining in testis sections of DMSO controls and MEKi-treated samples after 24 h of culture. DAPI (blue), pERK1/2 (green) and SOX9 (red). Arrows indicate pERK1/2 positive Sertoli cells. Scale bar represents 100 μm for 24 h samples or 500 μm for 72 h samples. Biological replicates; n = 4 testes per treatment. [file 12915_2024_2003_MOESM3_ESM.pdf]

Figure S2

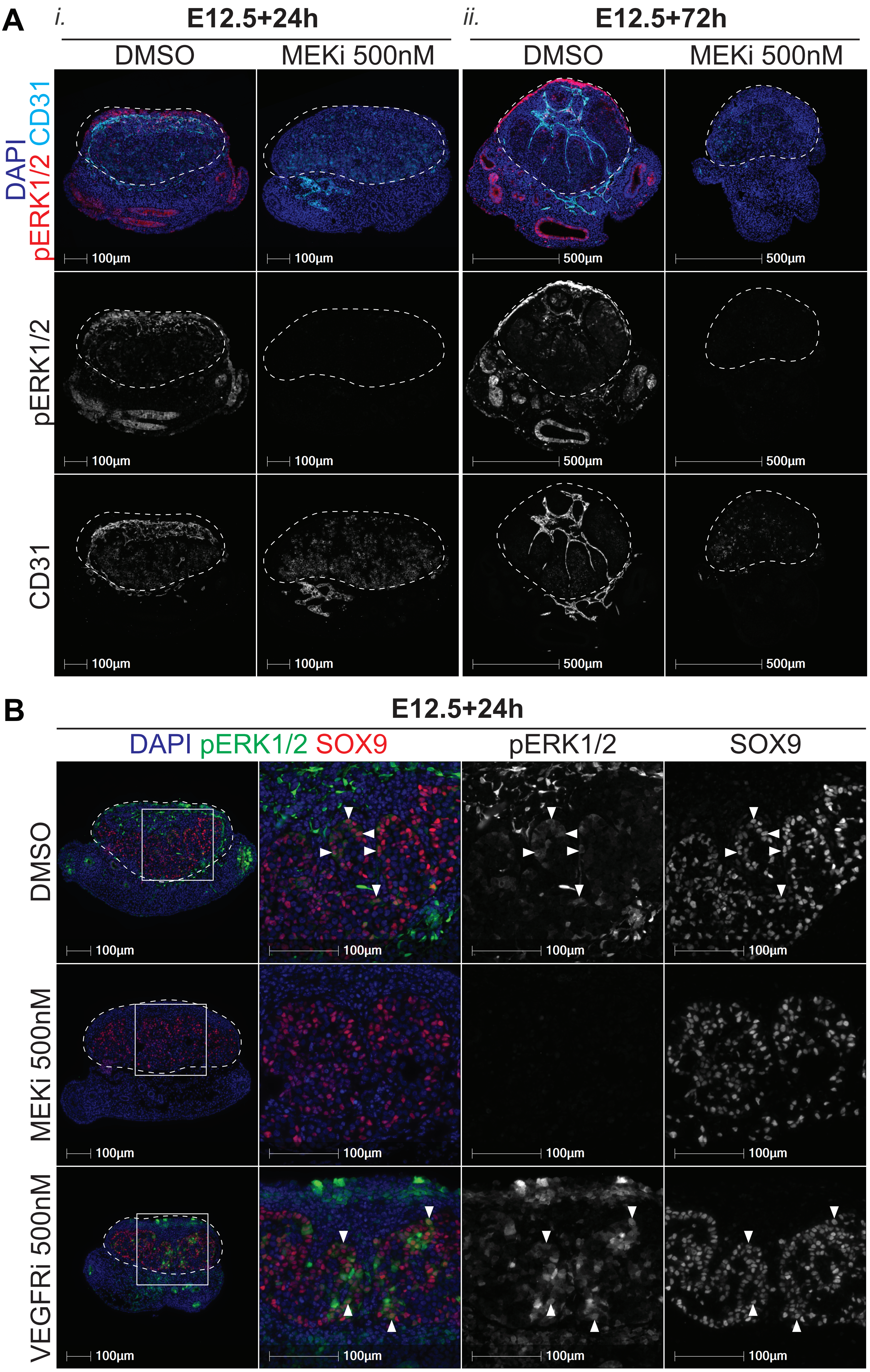

A
i.
E12.5+24h
ii.
E12.5+72h
DMSO
MEKi 500nM
DMSO
MEKi 500nM
DAPI pERK1/2 CD31
pERK1/2
CD31
100µm
500µm
B
E12.5+24h
DAPI pERK1/2 SOX9
pERK1/2
SOX9
DMSO
MEKi 500nM
VEGFRi 500nM
100µm

Additional file 3: Fig. S2. Immunofluorescent images of E12.5 testes cultured for 24 or 72h with DMSO or 500nM MEKi. A) Wide field images of sections from E12.5 testes cultured for 24 (i) or 72 h (ii) shown in Fig. 3. Images show DAPI (blue), pERK1/2 (red) and CD31 (endothelial cells and germ cells; cyan). B) IF images showing SOX9 and pERK1/2 double staining in testis sections of DMSO controls and MEKi-treated samples after 24h of culture. DAPI (blue), pERK1/2 (green) and SOX9 (red). Arrows indicate pERK1/2 positive Sertoli cells. Scale bar represents 100 μm for 24h samples or 500 μm for 72h samples. Biological replicates; n = 4 testes per treatment.
