# Supplementary material for: VEGF-dependent testicular vascularisation involves MEK1/2 signalling and the essential angiogenesis factors, SOX7 and SOX17
Source: BMC Biol. 2024 Oct 1;22:222. doi: 10.1186/s12915-024-02003-y (PMC11445939; doi:10.1186/s12915-024-02003-y)
Supplement: Supplementary file 8 — Additional file 8: Fig. S3. Epithelial cell marker, VE-cadherin, is lost in gonads treated with MEK1/2 inhibitor for 24 h. Immunofluorescence images showing VE-cadherin and CD31 double staining in gonad sections of DMSO controls and 500 nM MEKi or 500 nM VEGFRi-treated samples after 24 h of culture. DAPI (blue), VE-cadherin (red) and CD31 (cyan). Scale bar represents 500 μm for wide field images or 100 μm in digital zoom images. Biological replicates; n = 4 testes per treatment. Key: G – gonad, M – mesonephros. [file 12915_2024_2003_MOESM8_ESM.pdf]

Figure S3

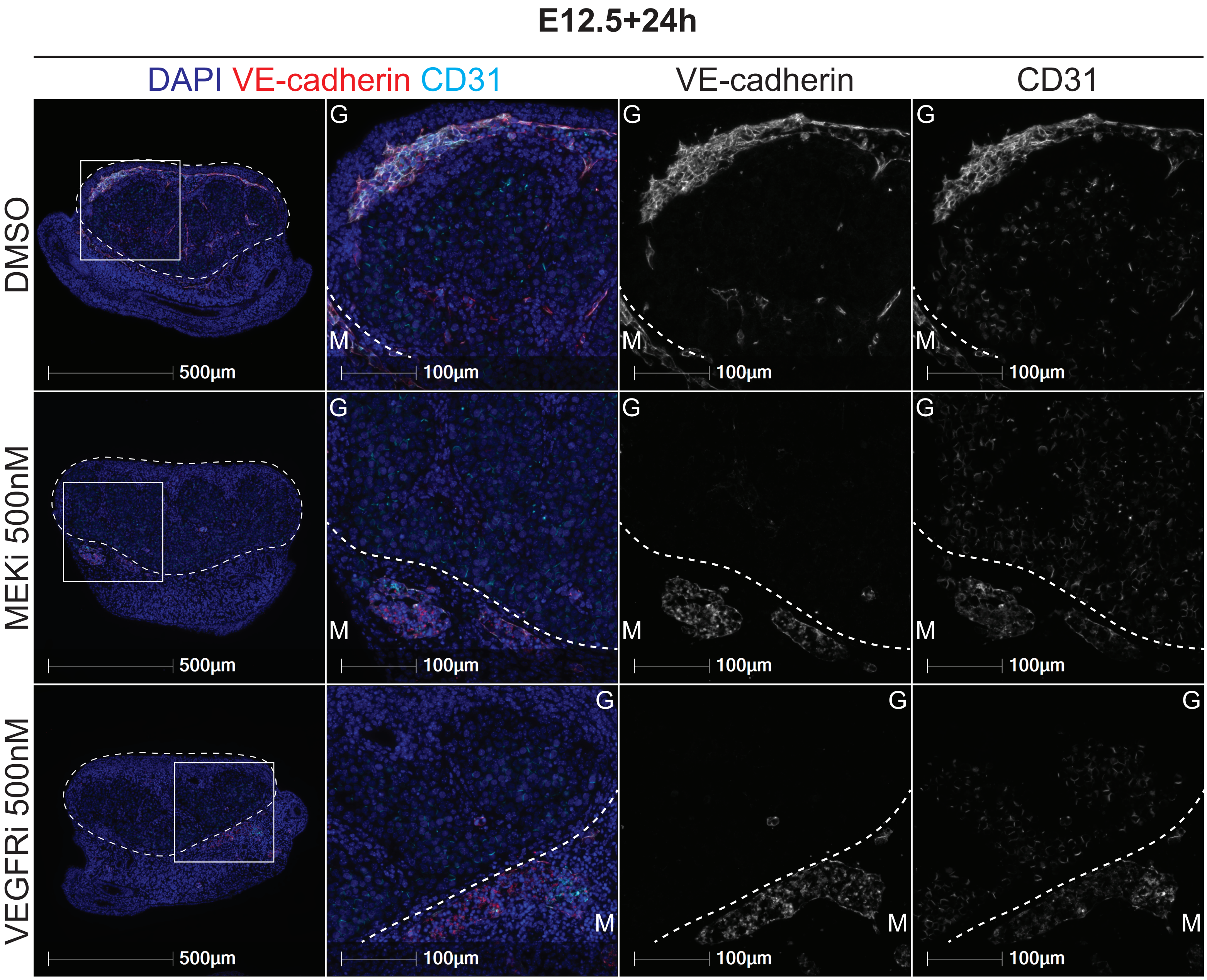

E12.5+24h
DAPI VE-cadherin CD31
VE-cadherin
CD31
DMSO
MEKi 500nM
VEGFRi 500nM
G
M
500µm
100µm

Additional file 8: Fig. S3. Epithelial cell marker, VE-cadherin, is lost in gonads treated with MEK1/2 inhibitor for 24h. Immunofluorescence images showing VE-cadherin and CD31 double staining in gonad sections of DMSO controls and 500nM MEKi or 500nM VEGFRi-treated samples after 24h of culture. DAPI (blue), VE-cadherin (red) and CD31 (cyan). Scale bar represents 500 μm for wide field images or 100 μm in digital zoom images. Biological replicates; n = 4 testes per treatment. Key: G – gonad, M – mesonephros.
