# Supplementary material for: VEGF-dependent testicular vascularisation involves MEK1/2 signalling and the essential angiogenesis factors, SOX7 and SOX17
Source: BMC Biol. 2024 Oct 1;22:222. doi: 10.1186/s12915-024-02003-y (PMC11445939; doi:10.1186/s12915-024-02003-y)
Supplement: Supplementary file 9 — Additional file 9: Fig. S4. Angiogenesis markers, SOX7 and SOX17, are expressed in endothelial cells. Immunofluorescent images of testes collected from E12.5 and E15.5 embryos and stained with DAPI (blue), SOX7 (red; A) or SOX17 (red; B) or SOX7/17 (red; C) and CD31 (endothelial cells and germ cells; cyan). Scale bar represents 500 μm in whole view images (first panel) or 100 μm in digital zoom images (right three panels). Biological replicates; n = 3 testes per stage. [file 12915_2024_2003_MOESM9_ESM.pdf]

Figure S4

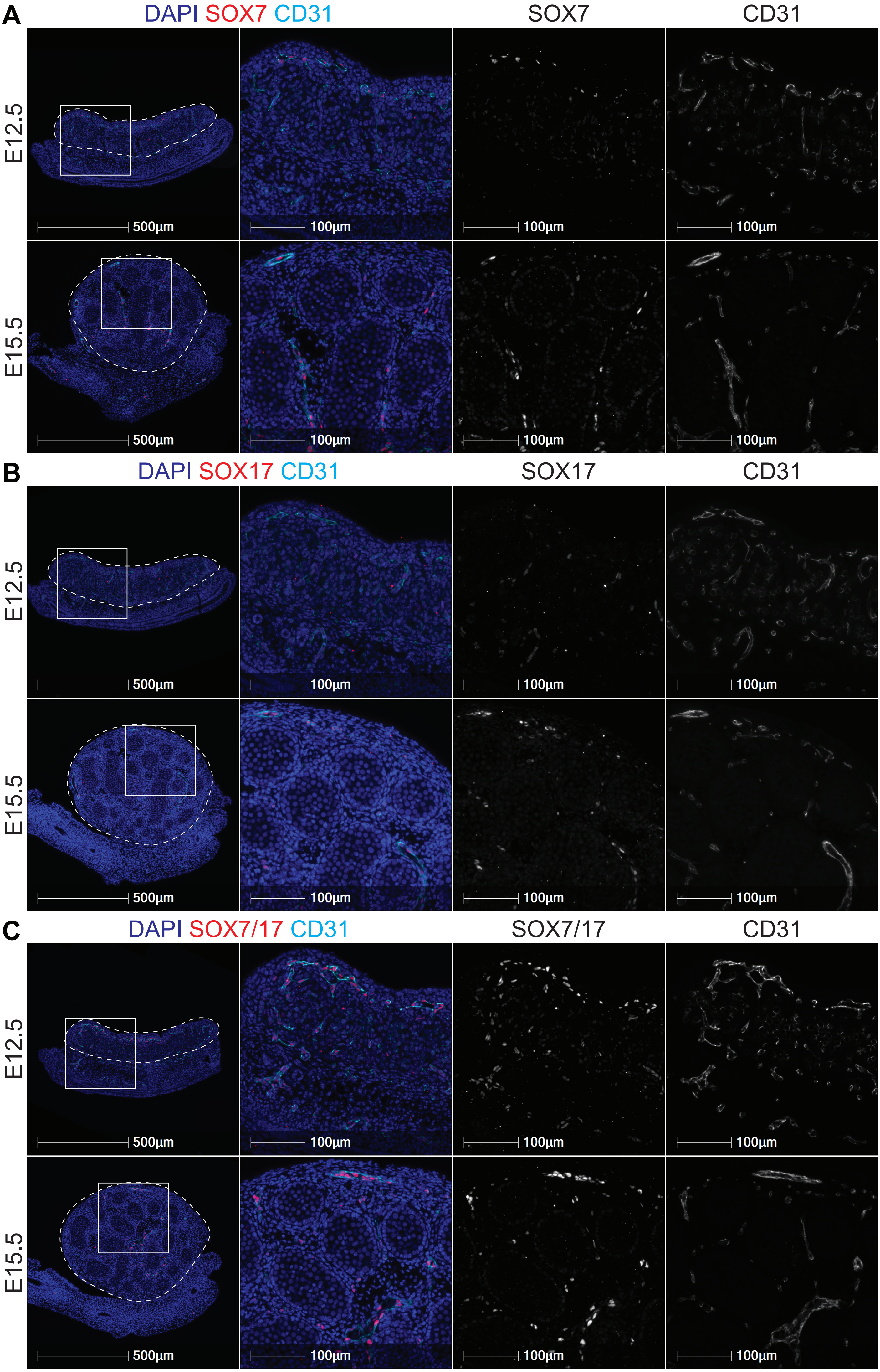
A
DAPI SOX7 CD31
SOX7
CD31
E12.5
E15.5
500µm
100µm
B
DAPI SOX17 CD31
SOX17
CD31
E12.5
E15.5
C
DAPI SOX7/17 CD31
SOX7/17
CD31
E12.5
E15.5

Additional file 9: Fig. S4. Angiogenesis markers, SOX7 and SOX17, are expressed in endothelial cells. Immunofluorescent images of testes collected from E12.5 and E15.5 embryos and stained with DAPI (blue), SOX7 (red; A) or SOX17 (red; B) or SOX7/17 (red; C) and CD31 (endothelial cells and germ cells; cyan). Scale bar represents 500 μm in whole view images (first panel) or 100 μm in digital zoom images (right three panels). Biological replicates; n = 3 testes per stage.
