# Supplementary material for: VEGF-dependent testicular vascularisation involves MEK1/2 signalling and the essential angiogenesis factors, SOX7 and SOX17
Source: BMC Biol. 2024 Oct 1;22:222. doi: 10.1186/s12915-024-02003-y (PMC11445939; doi:10.1186/s12915-024-02003-y)
Supplement: Supplementary file 10 — Additional file 10: Fig. S5. SOX7/17 is detected in endothelial cells in E12.5-E15.5 testes. Immunofluorescent imaging of testes collected from E12.5, E13.5, E14.5 and E15.5 embryos, stained with DAPI (blue), SOX7/17 (red) and CD31 (cyan). Arrows indicate SOX7/17 positive endothelial. Testes were obtained from embryos collected directly from pregnant females at E12.5, E13.5, E14.5 and E15.5. Scale bar represents 500 μm in whole view images (first panel) or 100 μm in digital zoom images (right three panels). Arrows indicate SOX7/17 positive endothelial cells. Biological replicates; n = 4 testes per stage. [file 12915_2024_2003_MOESM10_ESM.pdf]

Figure S5

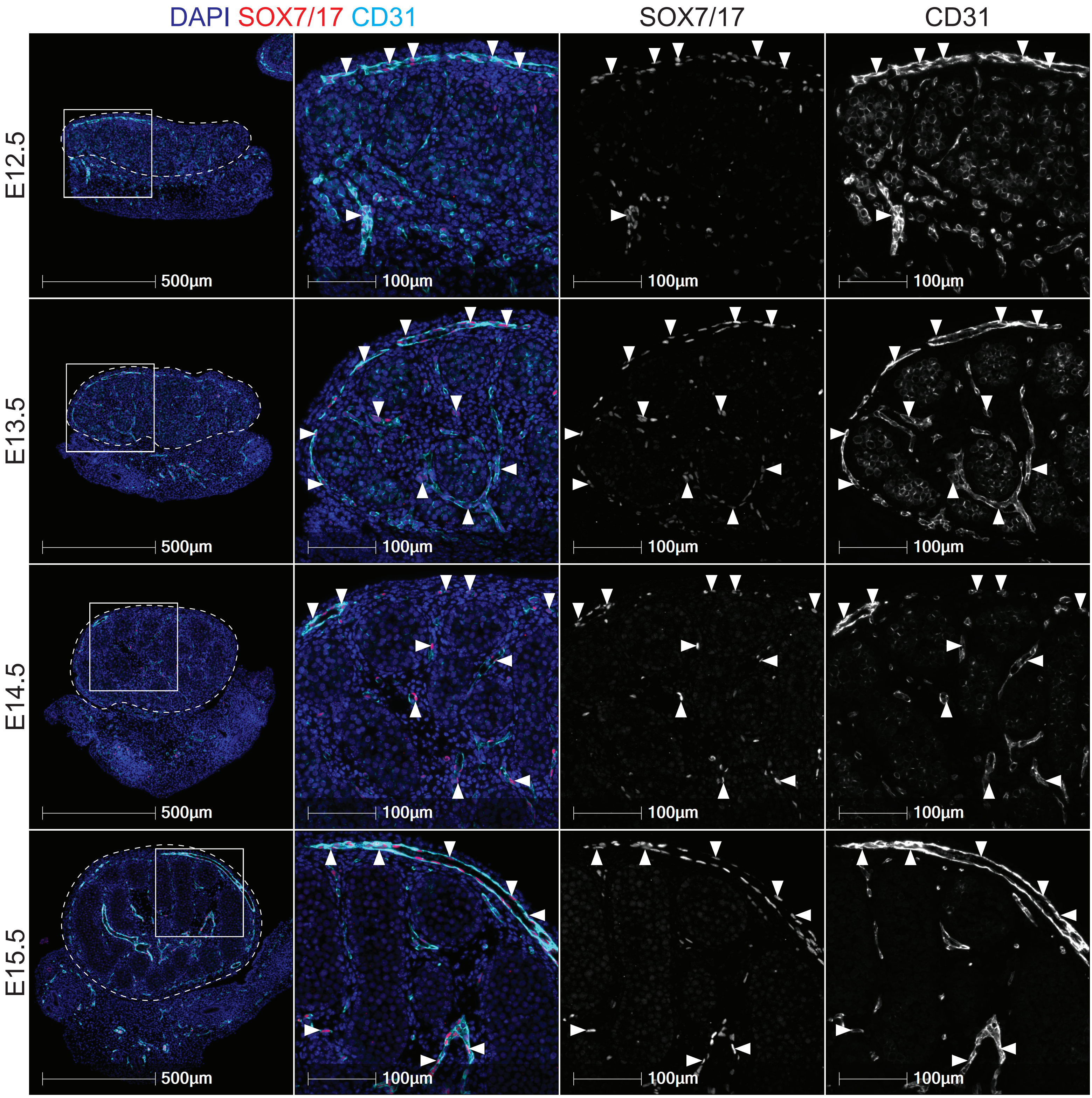

DAPI SOX7/17 CD31
SOX7/17
CD31
E12.5
E13.5
E14.5
E15.5
500µm
100µm

Additional file 10: Fig. S5. SOX7/17 is detected in endothelial cells in E12.5-E15.5 testes. Immunofluorescent imaging of testes collected from E12.5, E13.5, E14.5 and E15.5 embryos, stained with DAPI (blue), SOX7/17 (red) and CD31 (cyan). Arrows indicate SOX7/17 positive endothelial. Testes were obtained from embryos collected directly from pregnant females at E12.5, E13.5, E14.5 and E15.5. Scale bar represents 500 μm in whole view images (first panel) or 100 μm in digital zoom images (right three panels). Arrows indicate SOX7/17 positive endothelial cells. Biological replicates; n = 4 testes per stage.
