# Supplementary material for: VEGF-dependent testicular vascularisation involves MEK1/2 signalling and the essential angiogenesis factors, SOX7 and SOX17
Source: BMC Biol. 2024 Oct 1;22:222. doi: 10.1186/s12915-024-02003-y (PMC11445939; doi:10.1186/s12915-024-02003-y)
Supplement: Supplementary file 11 — Additional file 11: Fig. S6. Wide field images and higher power images of the gonad-mesonephric border of testis sections shown in Fig. 4. Immunofluorescent wide field images (A and C) or higher power images of the gonad-mesonephric border (B) of E12.5 testes cultured for 24 (A and B) or 72 h (C) with DMSO or 500 nM of MEKi stained with DAPI (blue), SOX7/17 (red) and CD31 (endothelial cells and germ cells; cyan). Scale bar represents 100 μm (A and B) or 500 μm (C). Biological replicates; n = 4 testes per treatment. Key: G = gonad, M = mesonephros. [file 12915_2024_2003_MOESM11_ESM.pdf]

Figure S6

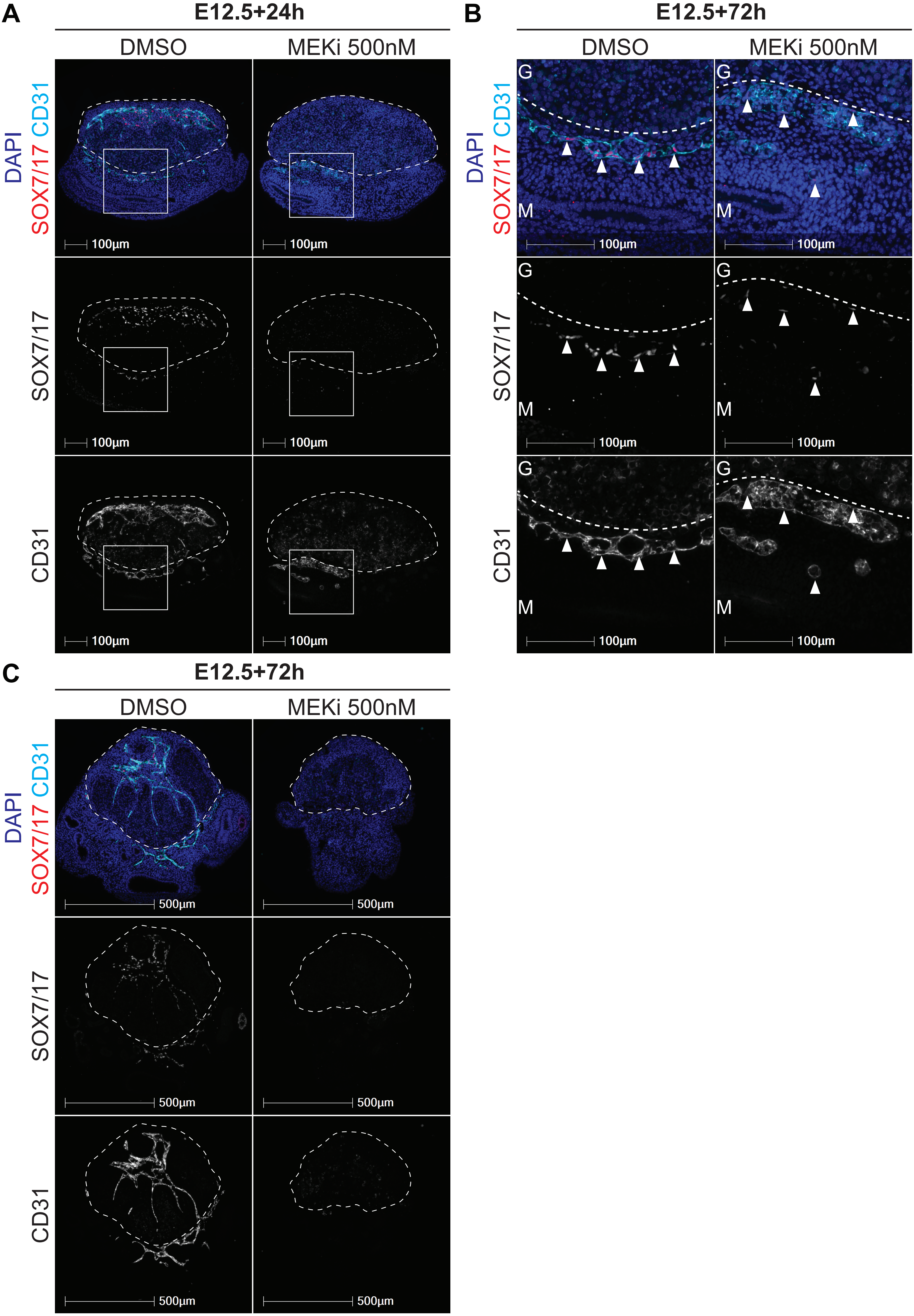
A
E12.5+24h
DMSO
MEKi 500nM
DAPI SOX7/17 CD31
SOX7/17
CD31
100µm
B
E12.5+72h
DMSO
MEKi 500nM
G
M
100µm
C
E12.5+72h
DMSO
MEKi 500nM
500µm

Additional file 11: Fig. S6. Wide field images and higher power images of the gonad-mesonephric border of testis sections shown in Fig. 4. Immunofluorescent wide field images (A and C) or higher power images of the gonad-mesonephric border (B) of sections of E12.5 testes cultured for 24 (A and B) or 72 h (C) with DMSO or 500nM of MEKi stained with DAPI (blue), SOX7/17 (red) and CD31 (endothelial cells and germ cells; cyan). Scale bar represents 100 μm (A and B) or 500 μm (C). Biological replicates; n = 4 testes per treatment. Key: G = gonad, M = mesonephros.
