# Supplementary material for: VEGF-dependent testicular vascularisation involves MEK1/2 signalling and the essential angiogenesis factors, SOX7 and SOX17
Source: BMC Biol. 2024 Oct 1;22:222. doi: 10.1186/s12915-024-02003-y (PMC11445939; doi:10.1186/s12915-024-02003-y)
Supplement: Supplementary file 16 — Additional file 16: Fig. S7. VEGFR inhibition reduces CD31 and pERK1/2 in developing testes. A) Wide field immunofluorescent images of E12.5 testes cultured for 24 h with DMSO or 100, 500 or 2500 nM of VEGFRi stained with DAPI (blue), pERK1/2 (red) and CD31 (endothelial cells and germ cells; cyan). Scale bars represent 100 μm. B) Immunofluorescent images of E12.5 testes cultured for 24 h with DMSO or 100, 500 or 2500 nM of Axitinib (a second VEGFR inhibitor) stained with DAPI (blue), pERK1/2 (red) and CD31 (cyan). Scale bars represent 500 μm (top panel) or 100 μm (bottom three panels). Arrows indicate endothelial cells and asterisks indicate pERK1/2 positive Sertoli cells. Biological replicates; n = 4 testes per stage. [file 12915_2024_2003_MOESM16_ESM.pdf]

Figure S7

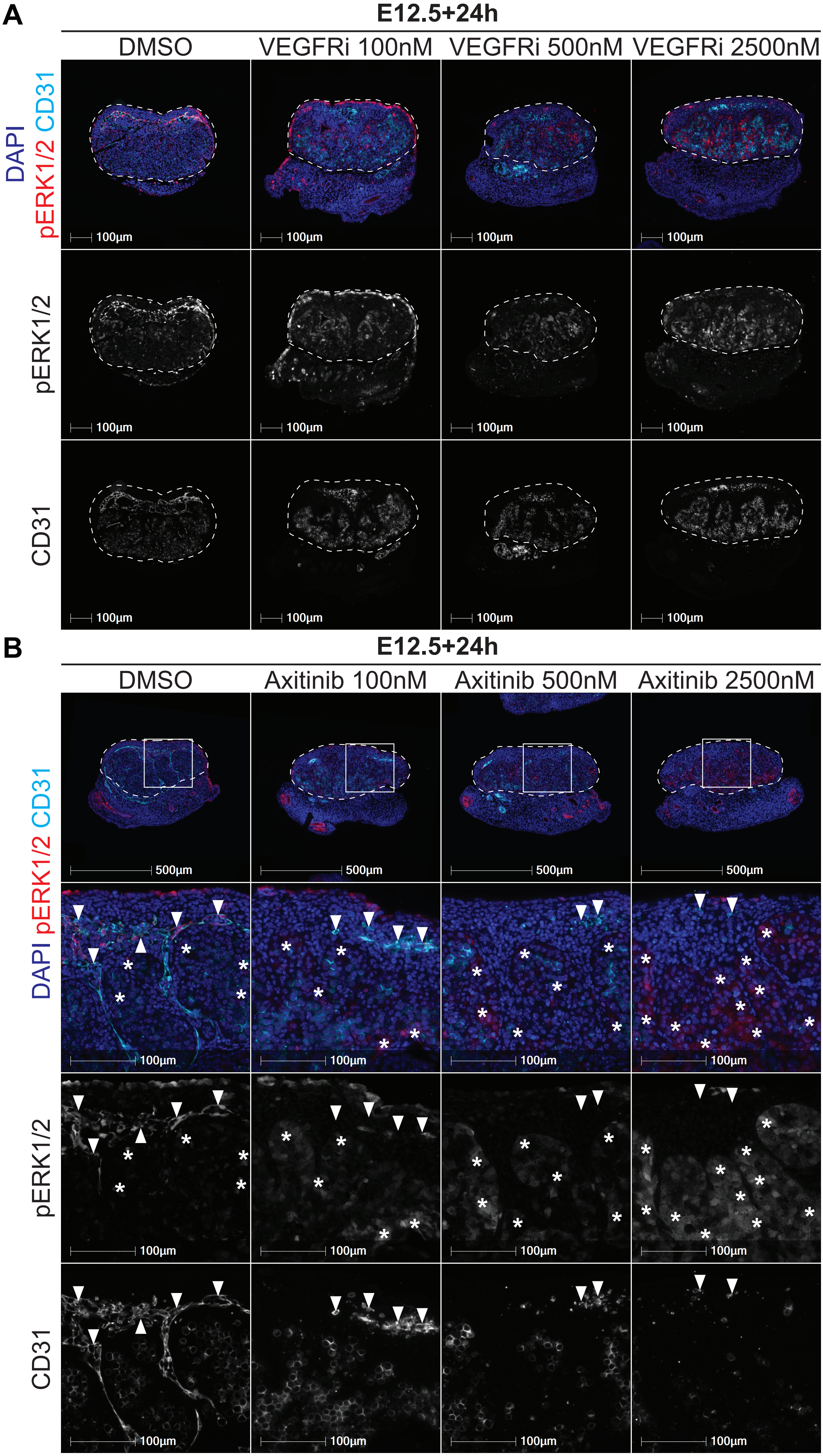

A
E12.5+24h
DMSO
VEGFRi 100nM
VEGFRi 500nM
VEGFRi 2500nM
DAPI pERK1/2 CD31
pERK1/2
CD31
100µm
B
E12.5+24h
DMSO
Axitinib 100nM
Axitinib 500nM
Axitinib 2500nM
DAPI pERK1/2 CD31
pERK1/2
CD31
500µm
100µm

Additional file 16: Fig. S7. VEGFR inhibition reduces CD31 and pERK1/2 in developing testes. A) Wide field immunofluorescent images of E12.5 testes cultured for 24h with DMSO or 100, 500 or 2500nM of VEGFRi stained with DAPI (blue), pERK1/2 (red) and CD31 (endothelial cells and germ cells; cyan). Scale bars represent 100 µm. B) Immunofluorescent images of E12.5 testes cultured for 24h with DMSO or 100, 500 or 2500nM of Axitinib (a second VEGFR inhibitor) stained with DAPI (blue), pERK1/2 (red) and CD31 (cyan). Scale bars represent 500 µm (top panel) or 100 µm (bottom three panels). Arrows indicate endothelial cells and asterisks indicate pERK1/2 positive Sertoli cells. Biological replicates; n = 4 testes per stage.
