# Supplementary material for: VEGF-dependent testicular vascularisation involves MEK1/2 signalling and the essential angiogenesis factors, SOX7 and SOX17
Source: BMC Biol. 2024 Oct 1;22:222. doi: 10.1186/s12915-024-02003-y (PMC11445939; doi:10.1186/s12915-024-02003-y)
Supplement: Supplementary file 21 — Additional file 21: Fig. S8. VEGFR inhibition reduces SOX7/17 in developing testes. A-B) Immunofluorescent wide field (A) or higher power images of the gonad-mesonephric border (B) of E12.5 testes cultured for 24 h with DMSO or 100, 500 or 2500 nM of VEGFRi stained with DAPI (blue), SOX7/17 (red) and CD31 (endothelial cells and germ cells; cyan). Scale bars represent 100 μm. C) Immunofluorescent imaging of E12.5 testes cultured for 24 h with DMSO or 100, 500 or 2500 nM of Axitinib (a second VEGFR inhibitor) stained with DAPI (blue), SOX7/17 (red) and CD31 (cyan). Scale bars represent 500 μm (top panel) or 100 μm (bottom three panels). Arrows indicate endothelial cells. Key: G = gonad, M = mesonephros. Biological replicates; n = 4. [file 12915_2024_2003_MOESM21_ESM.pdf]

Figure S8

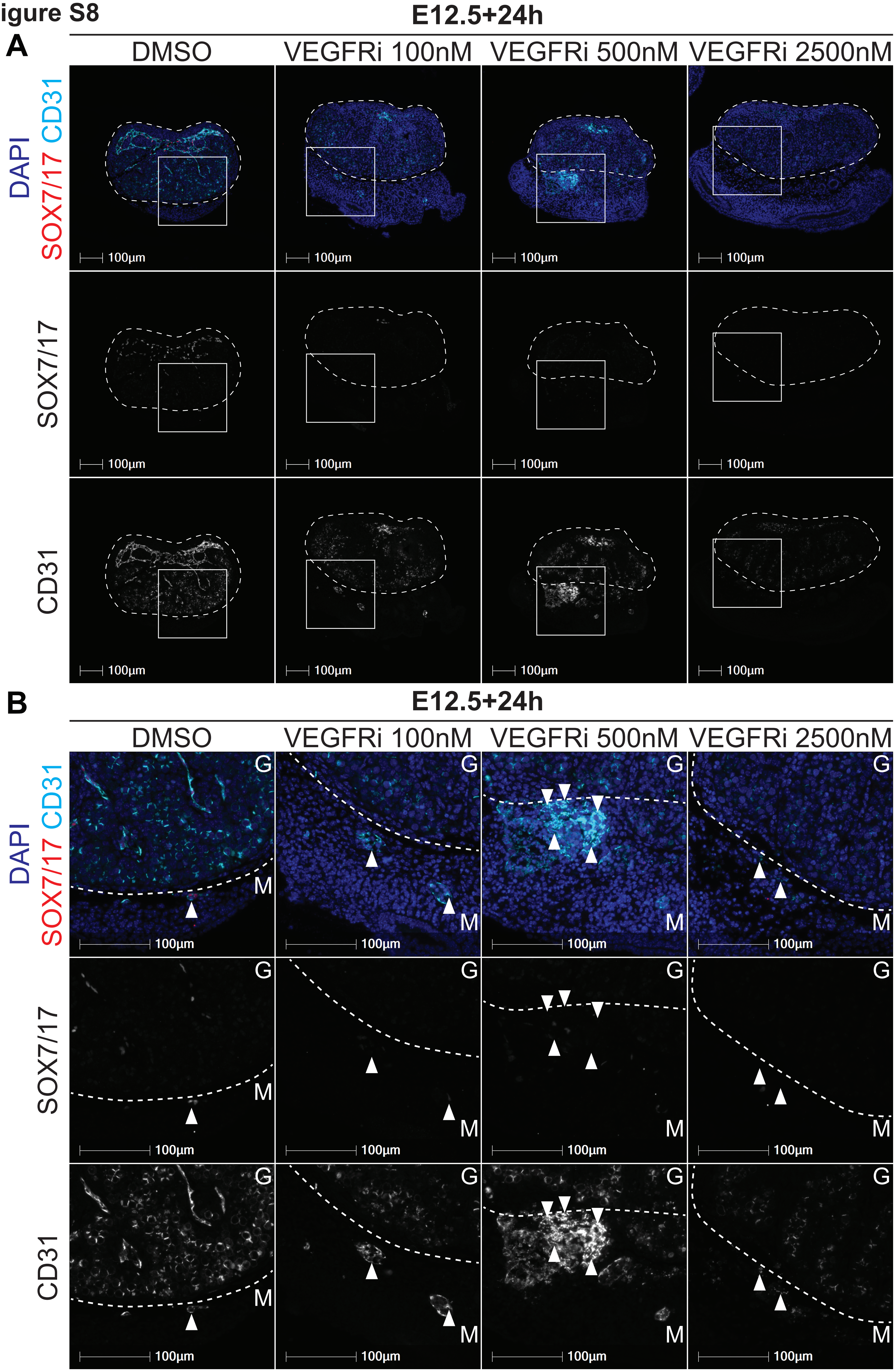
A
E12.5+24h
DMSO
VEGFRi 100nM
VEGFRi 500nM
VEGFRi 2500nM
DAPI SOX7/17 CD31
SOX7/17
CD31
100µm
B
E12.5+24h
DMSO
VEGFRi 100nM
VEGFRi 500nM
VEGFRi 2500nM
DAPI SOX7/17 CD31
SOX7/17
CD31
G
M
100µm

Figure S8 contintued

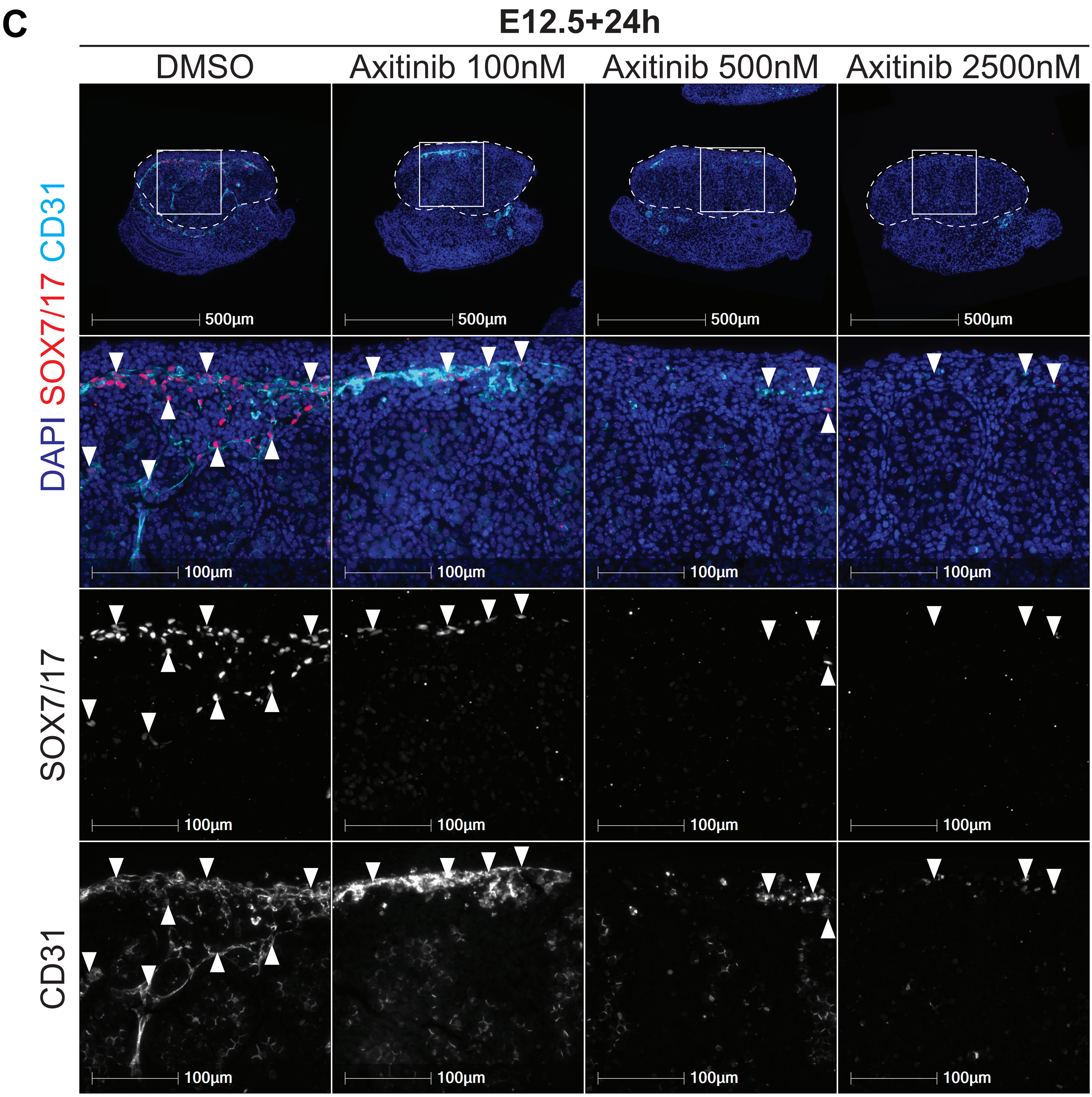

Additional file 21: Fig. S8. VEGFR inhibition reduces SOX7/17 in developing testes. A-B) Immunofluorescent wide field (A) or higher power images of the gonad-mesonephric border (B) of E12.5 testes cultured for 24h with DMSO or 100, 500 or 2500nM of VEGFRi stained with DAPI (blue), SOX7/17 (red) and CD31 (endothelial cells and germ cells; cyan). Scale bars represent 100 µm. C) Immunofluorescent imaging of E12.5 testes cultured for 24h with DMSO or 100, 500 or 2500nM of Axitinib (a second VEGFR inhibitor) stained with DAPI (blue), SOX7/17 (red) and CD31 (cyan). Scale bars represent 500 µm (top panel) or 100 µm (bottom three panels). Arrows indicate endothelial cells. Key: G = gonad, M = mesonephros. Biological replicates; n = 4.
