# Supplementary material for: VEGF-dependent testicular vascularisation involves MEK1/2 signalling and the essential angiogenesis factors, SOX7 and SOX17
Source: BMC Biol. 2024 Oct 1;22:222. doi: 10.1186/s12915-024-02003-y (PMC11445939; doi:10.1186/s12915-024-02003-y)
Supplement: Supplementary file 22 — Additional file 22. Fig. S9. VEGF inhibition prevents endothelial cell activation of pERK1/2 and SOX7/17 expression in the developing testes. Immunofluorescent images E12.5 testes cultured for with DMSO or 500 nM of VEGFRi for 72 h showing DAPI (blue), pERK1/2 (red; A), SOX7/17 (red; B) and CD31 (cyan). Scale bars represent 500 μm (whole view images) or 100 μm (digital zoom images). [file 12915_2024_2003_MOESM22_ESM.pdf]

Figure S9

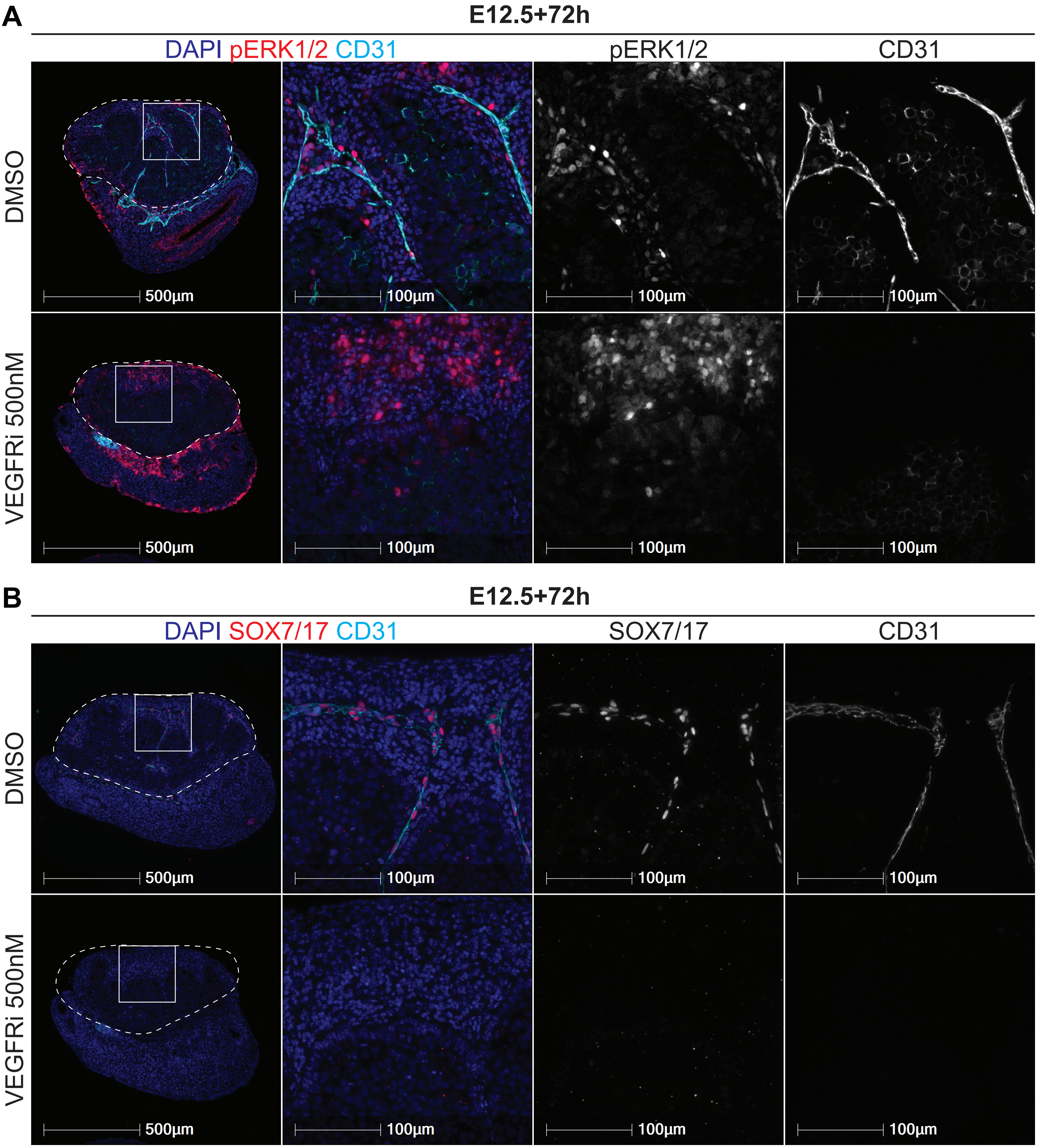
A
E12.5+72h
DAPI pERK1/2 CD31
pERK1/2
CD31
DMSO
VEGFRi 500nM
500µm
100µm
B
E12.5+72h
DAPI SOX7/17 CD31
SOX7/17
CD31
DMSO
VEGFRi 500nM
500µm
100µm

Additional file 22. Fig. S9. VEGF inhibition prevents endothelial cell activation of pERK1/2 and SOX7/17 expression in the developing testes. Immunofluorescent images E12.5 testes cultured for with DMSO or 500nM of VEGFRi for 72h showing DAPI (blue), pERK1/2 (red; A), SOX7/17 (red; B) and CD31 (cyan). Scale bars represent 500 μm (whole view images) or 100 μm (digital zoom images).
