# Supplementary material for: VEGF-dependent testicular vascularisation involves MEK1/2 signalling and the essential angiogenesis factors, SOX7 and SOX17
Source: BMC Biol. 2024 Oct 1;22:222. doi: 10.1186/s12915-024-02003-y (PMC11445939; doi:10.1186/s12915-024-02003-y)
Supplement: Supplementary file 27 — Additional file 27: Fig. S10. Endothelial cells are lost in E12.5 testes treated with MEK1/2 or VEGFR inhibitor. A) Immunofluorescent images of E12.5 testes cultured for with DMSO or 500 nM of MEKi or VEGFRi for 24 h stained for DAPI (blue), CD31 (green), cCaspase 3/9 (red) and EdU (cell proliferation; cyan). Scale bars represent 500 μm. B) Flow cytometric analysis of non-Sertoli somatic cell proliferation based on EdU incorporation in E12.5 testes cultured for 24 h with DMSO, 500 nM of MEKi or 100, 500, or 2500 nM of Axitinib. Biological replicates: In A; n = 4 per treatment, in B; n = 4–6. Statistics analysed using one-way ANOVA with Tukey’s multiple comparisons. Data represents mean ± SEM. Significance between DMSO and treatments; * < 0.05,**P < 0.01, ***P < 0.001, ****P < 0.0001. [file 12915_2024_2003_MOESM27_ESM.pdf]

Figure S10

A

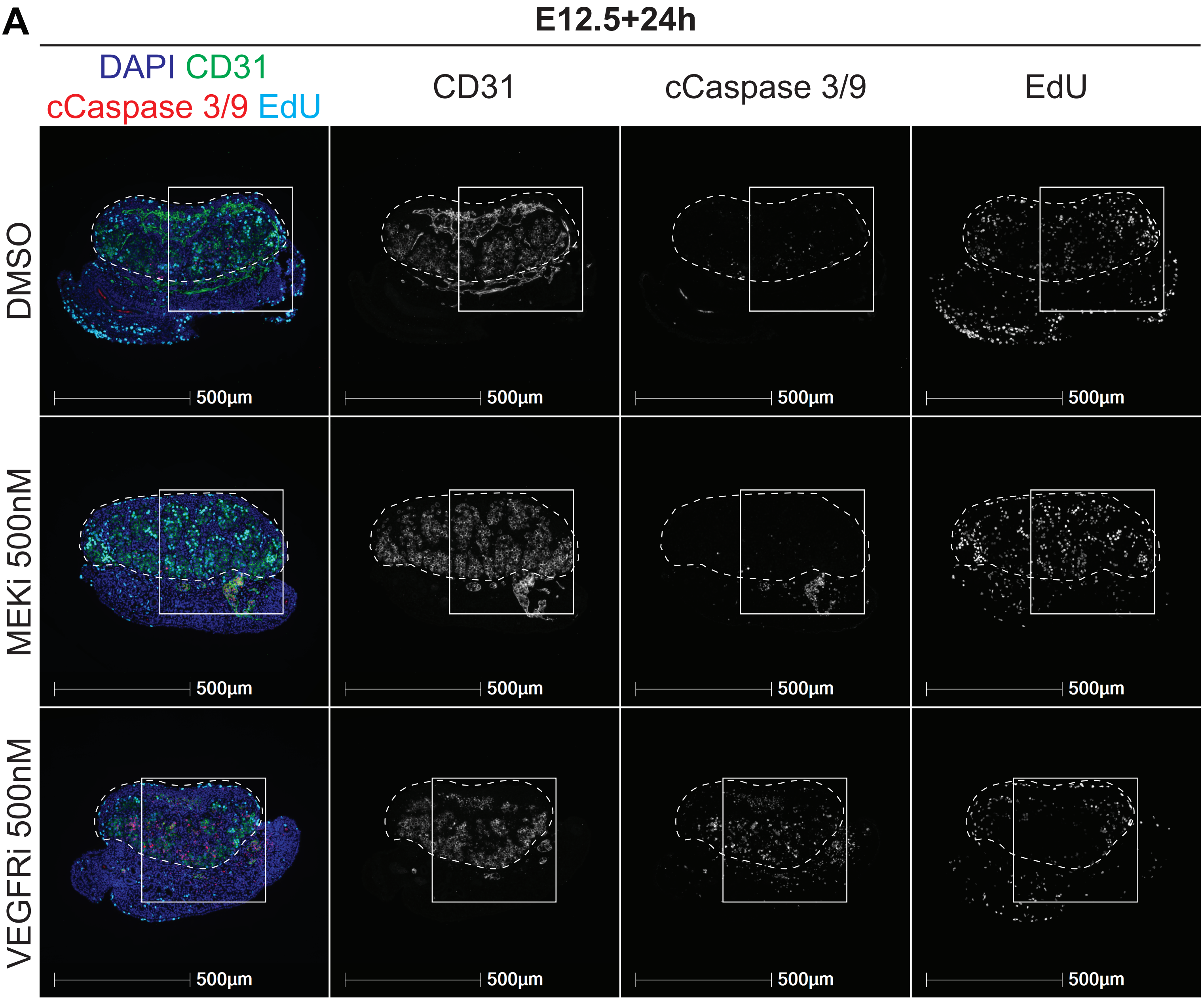

B

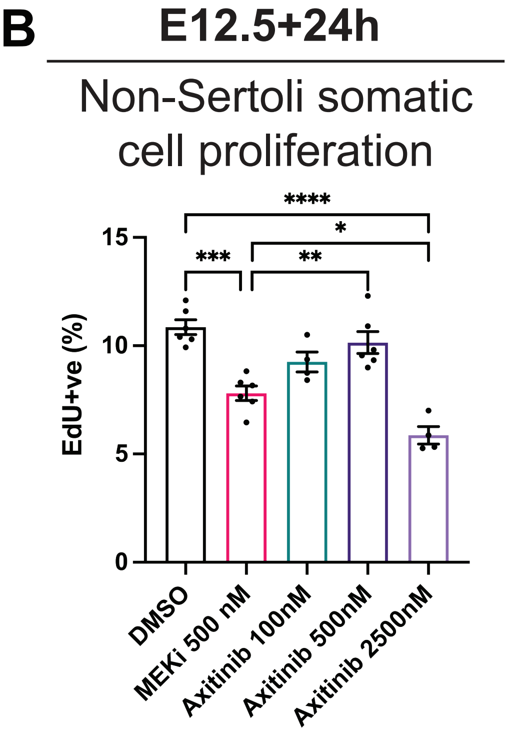

Additional file 27: Fig. S10. Endothelial cells are lost in E12.5 testes treated with MEK1/2 or VEGFR inhibitor. A) Immunofluorescent images of E12.5 testes cultured for with DMSO, or 500nM of MEKi or VEGFRi for 24h stained for DAPI (blue), CD31 (green), cCaspase 3/9 (red) and EdU (cell proliferation; cyan). Scale bars represent 500 µm. B) Flow cytometric analysis of non-Sertoli somatic cell proliferation based on EdU incorporation in E12.5 testes cultured for 24h with DMSO, 500nM of MEKi or 100, 500, or 2500nM of Axitinib. Biological replicates: In A; n = 4 per treatment, in B; n = 4–6. Statistics analysed using one-way ANOVA with Tukey's multiple comparisons. Data represents mean ± SEM. Significance between DMSO and treatments; * < 0.05, **P < 0.01, ***P < 0.001, ****P < 0.0001.
