# Supplementary material for: VEGF-dependent testicular vascularisation involves MEK1/2 signalling and the essential angiogenesis factors, SOX7 and SOX17
Source: BMC Biol. 2024 Oct 1;22:222. doi: 10.1186/s12915-024-02003-y (PMC11445939; doi:10.1186/s12915-024-02003-y)
Supplement: Supplementary file 28 — Additional file 28: Fig. S11. MEK1/2 signalling inhibition disrupts Sertoli cell proliferation and Sertoli cell localisation to the testis basement membrane. A) Flow cytometric analysis of E12.5 testes cultured with DMSO or 100, 500 or 2500 nM of Axitinib showing Sertoli cell proliferation based on EdU incorporation. B) HALO AI analysis of Sertoli cell organisation in E12.5 testes cultured with DMSO or 500 nM ofMEKi, VEGFRi or FGFRi. The first column of images shows representative staining of DAPI (blue), MVH (germ cells; green), SOX9 (Sertoli cells; red) and SMA (peritubular myoid cells; cyan). The second column of images demonstrates the region of interest (ROI) identified with HALO AI. The third column of images shows Sertoli cells selected based on SOX9 staining intensity. The last column shows the layers of Sertoli cells analysed using infiltration analysis. Biological replicates: A; n = 4–6. B; n = 3–9 per treatment. Statistics analysed using one-way ANOVA with Tukey’s multiple comparisons. Data represents mean ± SEM. Significance between DMSO and treatments; * < 0.05,**P < 0.01, ***P < 0.001, ****P < 0.0001. [file 12915_2024_2003_MOESM28_ESM.pdf]

Figure S11

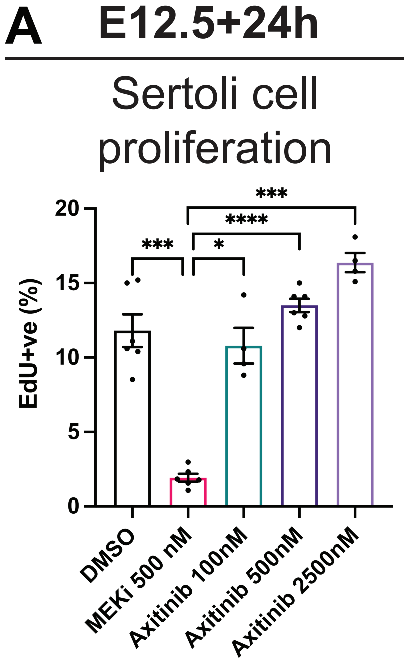
A
E12.5+24h
Sertoli cell proliferation
EdU+ve (%)
20
15
10
5
0
***
****
***
*
DMSO
MEKi 500 nM
Axitinib 100nM
Axitinib 500nM
Axitinib 2500nM

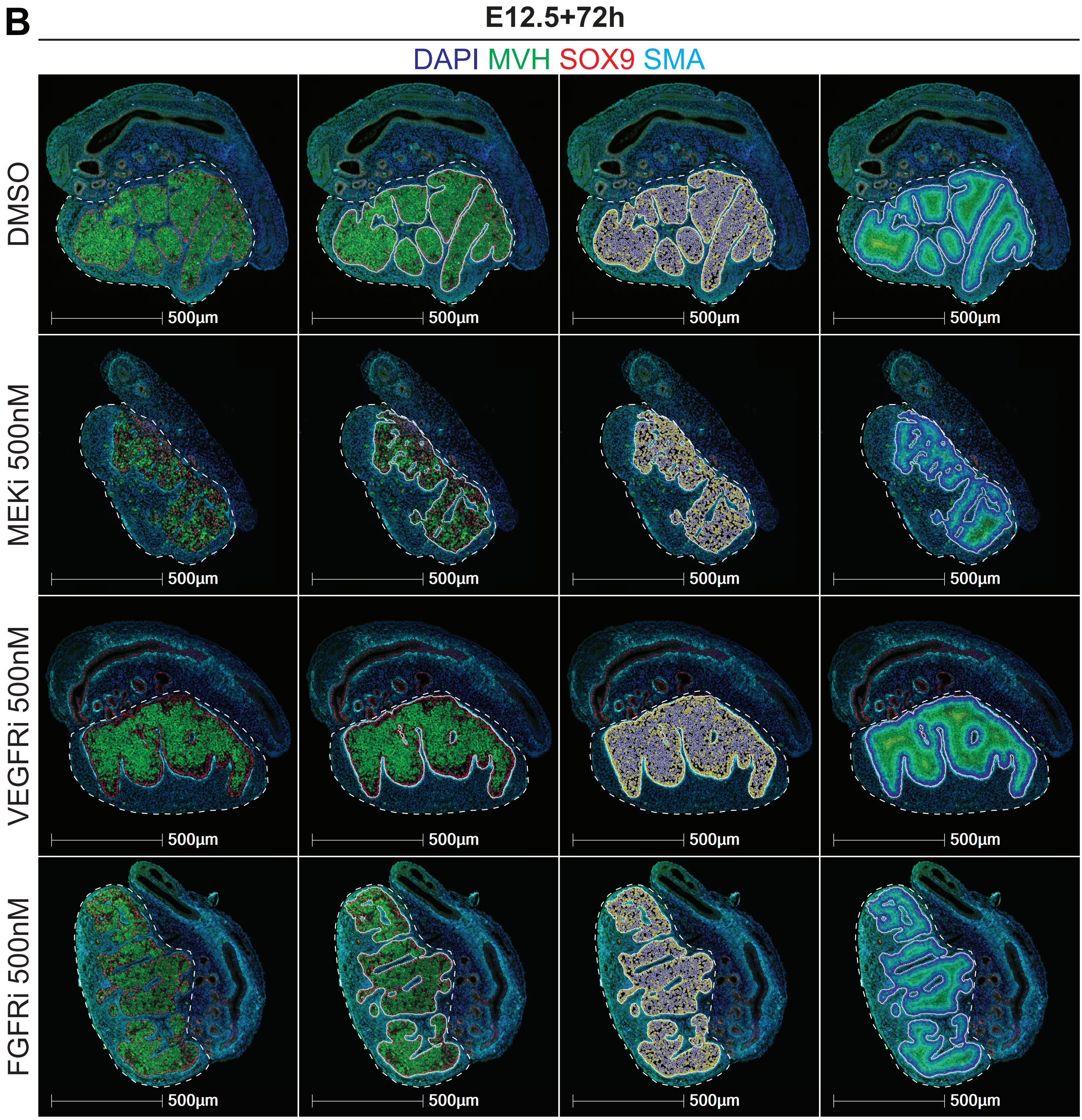
B
E12.5+72h
DAPI MVH SOX9 SMA
DMSO
MEKi 500nM
VEGFRi 500nM
FGFRi 500nM
500µm

Additional file 28: Fig. S11. MEK1/2 signalling inhibition disrupts Sertoli cell proliferation and Sertoli cell localisation to the testis basement membrane. A) Flow cytometric analysis of E12.5 testes cultured with DMSO or 100, 500 or 2500nM of Axitinib showing Sertoli cell proliferation based on EdU incorporation. B) HALO AI analysis of Sertoli cell organisation in E12.5 testes cultured with DMSO or 500nM of MEKi, VEGFRi or FGFRi. The first column of images shows representative staining of DAPI (blue), MVH (germ cells; green), SOX9 (Sertoli cells; red) and SMA (peritubular myoid cells; cyan). The second column of images demonstrates the region of interest (ROI) identified with HALO AI. The third column of images shows Sertoli cells selected based on SOX9 staining intensity. The last column shows the layers of Sertoli cells analysed using infiltration analysis. Biological replicates: A; n = 4–6. B; n = 3–9 per treatment. Statistics analysed using one-way ANOVA with Tukey's multiple comparisons. Data represents mean ± SEM. Significance between DMSO and treatments; * < 0.05, **P < 0.01, ***P < 0.001, ****P < 0.0001.
