# Supplementary material for: VEGF-dependent testicular vascularisation involves MEK1/2 signalling and the essential angiogenesis factors, SOX7 and SOX17
Source: BMC Biol. 2024 Oct 1;22:222. doi: 10.1186/s12915-024-02003-y (PMC11445939; doi:10.1186/s12915-024-02003-y)
Supplement: Supplementary file 29 — Additional file 29: Fig. S12. Gating strategy and controls for flow cytometric analysis. A = Area, W = Width. A) Single cells were identified based on propidium iodide (PI) width vs area plots. Somatic and germ cells were separated based on MVH expression with germ cells identified as MVH positive and somatic cells identified as MVH negative. The somatic cell population was separated into SOX9 expressing Sertoli cells and SOX9 negative non-Sertoli somatic cells. Cell cycle analysis was performed In the Sertoli cell and non-Sertoli somatic cell populations based on EdU incorporation to identify cells in S-phase, with EdU negative cells in G0/G1 and G2/M identified according to DNA content determined by PI staining intensity (cells in G0/G1 have low PI intensity / 2N DNA content while cells in G2/M have high PI intensity / 4N DNA content). B) Appropriate controls were used to set gating including mesonephric cells to identify MVH negative cells (i), ovarian cells to identify SOX9 negative cells (ii) and cells not treated with EdU were used to identify the EdU negative population (iii). [file 12915_2024_2003_MOESM29_ESM.pdf]

Figure S12

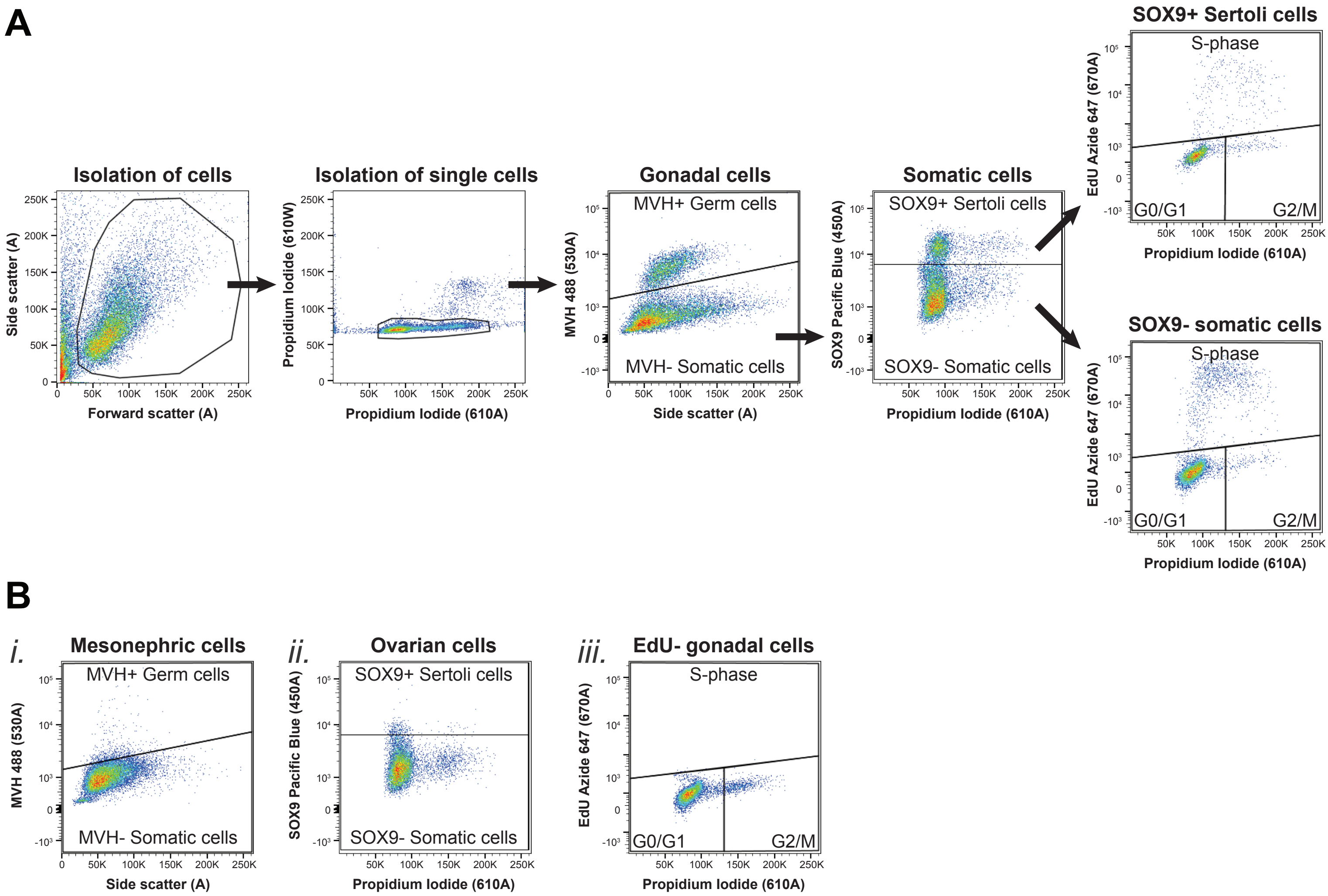
A
Isolation of cells
Forward scatter (A)
Side scatter (A)
Isolation of single cells
Propidium Iodide (610A)
Propidium Iodide (610W)
Gonadal cells
MVH+ Germ cells
MVH- Somatic cells
Side scatter (A)
MVH 488 (530A)
Somatic cells
SOX9+ Sertoli cells
SOX9- Somatic cells
Propidium Iodide (610A)
SOX9 Pacific Blue (450A)
SOX9+ Sertoli cells
S-phase
G0/G1
G2/M
Propidium Iodide (610A)
EdU Azide 647 (670A)
SOX9- somatic cells
S-phase
G0/G1
G2/M
Propidium Iodide (610A)
EdU Azide 647 (670A)
B
i.
Mesonephric cells
MVH+ Germ cells
MVH- Somatic cells
Side scatter (A)
MVH 488 (530A)
ii.
Ovarian cells
SOX9+ Sertoli cells
SOX9- Somatic cells
Propidium Iodide (610A)
SOX9 Pacific Blue (450A)
iii.
EdU- gonadal cells
S-phase
G0/G1
G2/M
Propidium Iodide (610A)
EdU Azide 647 (670A)

Additional file 29: Fig. S12. Gating strategy and controls for flow cytometric analysis. A = Area, W = Width. A) Single cells were identified based on propidium iodide (PI) width vs area plots. Somatic and germ cells were separated based on MVH expression with germ cells identified as MVH positive and somatic cells identified as MVH negative. The somatic cell population was separated into SOX9 expressing Sertoli cells and SOX9 negative non-Sertoli somatic cells. Cell cycle analysis was performed In the Sertoli cell and non-Sertoli somatic cell populations based on EdU incorporation to identify cells in S-phase, with EdU negative cells in G0/G1 and G2/M identified according to DNA content determined by PI staining intensity (cells in G0/G1 have low PI intensity / 2N DNA content while cells in G2/M have high PI intensity / 4N DNA content). B) Appropriate controls were used to set gating including mesonephric cells to identify MVH negative cells (i), ovarian cells to identify SOX9 negative cells (ii) and cells not treated with EdU were used to identify the EdU negative population (iii).
